# In Silico Development of a Multi-Epitope Subunit Vaccine against Bluetongue Virus in *Ovis aries* Using Immunoinformatics

**DOI:** 10.3390/pathogens13110944

**Published:** 2024-10-29

**Authors:** Priyansha Raj Sinha, Shubhada R. Hegde, Ruchika Mittal, Chikkamagaluru Chandrashekhar Jagat, Ullas Gowda, Rathna Chandrashekhar, Gayathri Muthaiah, Samer Shamshad, Mohammed Mudassar Chanda, Vishweshwar Ganji, Kalyani Putty, Divakar Hemadri

**Affiliations:** 1 Institute of Bioinformatics and Applied Biotechnology (IBAB), Bengaluru 560100, India; 2 School of Biosciences, Chanakya University, Bengaluru 562110, India; 3 ICAR—National Institute of Veterinary Epidemiology and Disease Informatics, Bengaluru 560064, India; 4 Department of Veterinary Biotechnology, CoVsc, PVNRTVU, Hyderabad 500030, India

**Keywords:** bluetongue virus, epitopes, subunit vaccine, *Ovis aries*

## Abstract

The bluetongue virus (BTV), transmitted by biting midges, poses a significant threat to livestock globally. This orbivirus induces bluetongue disease, leading to substantial economic losses in the agricultural sector. The current control measures have limitations, necessitating the development of novel, efficient vaccines. In this study, an immunoinformatics approach is employed to design a multi-epitope subunit vaccine for *Ovis aries* targeting six BTV serotypes. Focusing on the VP2 capsid protein, the vaccine incorporates B-cell, helper-T lymphocytes (HTL), and cytotoxic T-cell lymphocytes (CTL) epitopes. Molecular docking reveals stable interactions with TLR2 and TLR4 receptors, suggesting the stability of the complex, indicating the potential viability of the multi-epitope vaccine. The computational approach offers a rapid and tailored strategy for vaccine development, highlighting potential efficacy and safety against BTV outbreaks. This work contributes to understanding BTV and presents a promising avenue for effective control.

## 1. Introduction

Bluetongue (BT) is an infectious and non-contagious viral disease affecting domestic and wild ruminants. It is caused by the non-enveloped icosahedral bluetongue virus (BTV), which belongs to the *Orbivirus* genus of the family *Sedoreoviridae* [1]. The virus possesses ten linear double-stranded RNA genome segments (Seg-1 to Seg-10) [2,3] that are responsible for encoding a total of seven structural proteins (VP1–VP7) and five non-structural proteins (NS1, NS2, NS3/NS3a, NS4, and NS5) [4,5] that contain antigenic epitopes. Both cellular and humoral immunity contribute to protection against BTV infection [6,7], and an effective vaccine should, therefore, aim to induce both [8]. Various vaccination approaches have been explored to combat the impact of BT on animal health and agriculture. Traditional inactivated vaccines, while safer, face limitations in providing cross-protection among different BTV serotypes and lack the ability to differentiate infected from vaccinated animals [9]. Live-attenuated vaccines have been historically used, but concerns about teratogenicity, reversion to virulence, and potential reassortment events with wild-type viruses pose risks [10]. Viral vector vaccines aim to stimulate innate immunity, but studies in ruminants show only partial protection, with detectable viral replication in some cases [11]. Despite the available options, limitations persist, prompting the exploration of different strategies. Recent computational advances in immunoinformatics offer a promising strategy for predicting antigenic epitopes with translational implications [12,13].

Keeping this in mind, in this study, we employed an immunoinformatics in silico approach to construct a multi-epitope-based subunit vaccine comprising B-cell epitopes, helper-T lymphocytes (HTL) epitopes, and cytotoxic T-cell lymphocytes (CTL) epitopes for six BTV serotypes (4, 10, 11, 17, 20, and 24) within antigenic group A [14]. These serotypes, characterized by cross-neutralization based on genome segment 2 expressing VP2, exhibit inter-serotype neutralization. Specifically, serotypes 4, 20, and 17 demonstrate strong cross-neutralization among themselves [15]. As sheep are recognized as the most susceptible hosts for BT [16,17], we aimed to develop both monovalent subunit vaccines for individual serotypes and a hexavalent vaccine for comprehensive immunization against all six serotypes in *Ovis aries* (sheep). TLR2 and TLR4, expressed on the cell surface, recognize viral proteins on the virion, inducing inflammatory cytokine production [18,19]. Despite their primary function in bacterial recognition, particularly, TLR2 and TLR4 are key to mediating innate immune responses to specific viral pathogens, targeting viral envelope proteins in the extracellular milieu [20]. Therefore, molecular docking and a subsequent molecular dynamics (MD) simulation were employed to evaluate vaccine binding affinity for TLR2 and TLR4, assessing its stability and related interactions.

## 2. Methods

### 2.1. Sequence Retrieval and Alignment

The amino acid FASTA sequences (South African reference), corresponding to the VP2 proteins of six BTV serotypes, were obtained from the UniProt database (https://www.uniprot.org/, accessed on 11 September 2023) [21] and aligned using the MUSCLE alignment tool [22]. The accession IDs for serotypes 4, 10, 11, 17, 20, and 24 are Q2VEZ8, A0PCN7, A0PCN8, A0PCP4, A0PCP7, and A0PCQ1, respectively. A consensus sequence for the VP2 protein for all six serotypes was generated using Jalview [23]. The tertiary structure of TLR2 (Uniprot id: W5Q0A3) and TLR4 (Uniprot id: W5P706) were retrieved from the Alphafold database. The Antigenic Peptide Prediction tool (http://imed.med.ucm.es/Tools/antigenic.pl, accessed on 15 September 2023) was employed to estimate the average antigenic propensities of the individual VP2 proteins, which utilizes the Kolaskar and Tongaonkar method and has an accuracy of 75% [24]. Subsequently, the retrieved protein sequences underwent a prediction of antigenic epitopes, focusing on both B-cell and T-cell antigens. The objective was to boost both humoral and cell-mediated immunity.

### 2.2. B-Cell Epitope Prediction

B cells, also known as B lymphocytes or bursa-derived cells, are crucial components of the adaptive immune response, playing a central role in providing humoral immunity in mammals [25]. B-cell epitope prediction was conducted using four different servers. (1) The first tool is Ellipro (http://tools.iedb.org/ellipro/, accessed on 29 September 2023) [26]. ElliPro predicts antibody epitopes in protein antigens using their three-dimensional structure. It employs a modified version of Thornton’s method to identify continuous epitopes in protein regions [27]. Therefore, to provide a tertiary structure as an input, VP2 proteins were modeled using the Robetta server (https://robetta.bakerlab.org/, accessed on 25 Septembber 2023) [28]. (2) The second tool is SVMTriP (http://sysbio.unl.edu/SVMTriP/, accessed on 30 September 2023). SVMTriP utilizes a support vector machine (SVM) to predict linear antigenic epitopes, combining Tri-peptide similarity and propensity scores for enhanced accuracy [29]. (3) The third tool is ABCPred (www.imtech.res.in/raghava/abcpred/, accessed on 5 October 2023), which uses a recurrent neural network (RNN) with propensity scales for amino acids to predict B-cell epitopes based on their physico-chemical properties [30]. (4) BcePred (http://www.imtech.res.in/raghava/bcepred/, accessed on 10 October 2023) predicts B-cell epitopes using four amino acid properties, namely hydrophilicity, flexibility, accessibility, and polarity, to enhance prediction accuracy in antigenic sequences [31].

### 2.3. HTL and CTL Epitope Prediction

The existing T-cell epitope prediction servers lack the incorporation of ovine MHC I and MHC II alleles. As an alternative, bovine alleles were selected for the specific prediction of MHC I and MHC II allele-associated epitopes. To refine the selection process for predicting HTL and CTL epitopes in sheep, we employed multiple bovine leukocyte antigen (BoLA) alleles available on the HTL and CTL prediction servers. The sequences of these BoLA alleles were retrieved from the IPD-MHC Database (https://www.ebi.ac.uk/ipd/mhc/, accessed on 20 October 2023) [32]. To ensure relevance to *Ovis aries*, we performed a BLASTP search using these BoLA sequences, with *Ovis aries* set as the target organism. Only those BoLA alleles that exhibited greater than 90% coverage and identity with ovine MHC sequences in the BLASTP results were selected. This approach ensured a tailored prediction of T-cell epitopes for sheep.

HTL epitope prediction was carried out utilizing the NetMHCIIpan—2.1 server (https://services.healthtech.dtu.dk/services/NetMHCIIpan-2.1/, accessed on 24 October 2023), employing selected MHC II alleles. The binding threshold (IC_50_ value) was set at 50. Epitope affinities were evaluated by considering the top prediction score and a percent rank below 0.5 [33]. Simultaneously, the prediction of MHC I-specific CTL epitopes utilized the NetMHCpan—4.1 server (https://services.healthtech.dtu.dk/services/NetMHCpan-4.1/, accessed on 27 October 2023), employing artificial neural networks for the peptide-binding prediction [34]. Epitopes exhibiting strong binding affinity, characterized by a predicted %Rank below 0.5, were considered.

### 2.4. Analysis of Epitope Antigenicity, Allergenicity, and Toxicity

Prior to designing the subunit vaccine, we evaluated the selected epitopes for their antigenic, allergenic, and toxic attributes. The epitope antigenicity was assessed using VaxiJen 2.0 (target organism: “virus”, threshold: default), which uses auto cross-covariance (ACC) for the classification of proteins based on physicochemical properties [35]. AllerTOP 2.0 was employed for predicting epitope allergenicity, which utilizes ACC transformation and five amino acid descriptors, applying the k-nearest neighbor algorithm (kNN, k = 1) with a training set of known allergens and non-allergens [36]. The ToxinPred server (threshold SVM: default) predicted the toxicity profile of vaccine-candidate epitopes based on dipeptide composition, achieving an accuracy of approximately 90% [37]. Only epitopes meeting non-toxic, non-allergenic, and antigenic criteria were selected for inclusion in the vaccine construct.

### 2.5. Subunit Vaccine Construction and Physicochemical Property Assessment

For the construction of the vaccine, all the selected B-cell, CTL, and HTL epitopes were assembled and connected using a suitable adjuvant, linkers, and His-tag. Linkers allow for optimal flexibility to the amino acid residues, allowing them to fold into favorable conformations [13]. To join B-cell, CTL, and HTL epitopes, KK, AAY, and GPGPG linkers were used, respectively. An adjuvant is used to enhance the vaccine’s antigenicity. Therefore, a TLR2 and TLR4 agonist, Beta defensin 3 (Uniprot ID-Q5U7J2) [38] was added as an adjuvant with the help of the EAAAK linker at the N-terminal of the vaccine construct. Beta defensin 3 has antimicrobial activity and plays a crucial role in immune responses, specifically in chemotaxis. It has been linked to the chemotaxis of immature dendritic cells and T cells by interacting with CCR6 and promoting the chemotaxis of monocytes by interacting with CCR2 [39]. The His-tag (6 H) was added at the C-terminal of the construct. Subsequently, the antigenicity and allergenicity of all seven vaccine constructs (six serotype specific and one consensus) were evaluated using the VaxiJen 2.0 server and the AllerTOP 2.0 server, respectively.

We further analyzed the physiochemical properties of the vaccine constructs using ExPASy ProtParam (https://web.expasy.org/protparam/, accessed on 15 November 2023). The tool calculates essential physico-chemical properties from a protein sequence and pK value, including the molecular weight, pI, composition, extinction coefficient, half-life, instability index, aliphatic index, and grand average of hydropathicity. It estimates extinction coefficients, predicts in vivo half-life based on the N-end rule, assesses stability through the instability index, and evaluates aliphatic side-chain volume using the aliphatic index [40,41].

### 2.6. Secondary and Tertiary Structure Modelling

The secondary structure prediction of the vaccine constructs was conducted using the PSIPRED server (http://bioinf.cs.ucl.ac.uk/psipred/, accessed on 21 November 2023). It utilizes two neural networks analyzing the PSI-BLAST (Position-Specific Iterated–BLAST) output. With a robust cross-validation approach, PSIPRED 4 attains an average Q3 score of 84.2% [42]. The tertiary structure prediction of the constructs was predicted through the Robetta server (https://robetta.bakerlab.org/, accessed on 24 November 2023) using the RoseTTAFold method. Robetta, utilizing the Rosetta software, employs comparative modeling, using confident matches to known structures as templates, and de novo structure prediction with the Rosetta fragment insertion method. This involves generating local conformation fragments from the PDB, assembling models through fragment insertion, and favoring protein-like features via a scoring function [28].

### 2.7. Tertiary Structure Refinement and Validation

The 3D models obtained from the Robetta server were subjected to refinement using the GalaxyRefine server (https://galaxy.seoklab.org/cgi-bin/submit.cgi?type=REFINE, accessed on 4 December 2023). GalaxyRefine begins by rebuilding side chains, placing high-probability rotamers layer by layer, and resolving clashes. It then refines the model through two relaxation methods, employing mild and aggressive strategies based on repetitive short molecular dynamics simulations [43]. Tertiary structure validation utilized the Swiss-Model structure assessment server (https://swissmodel.expasy.org/assess, accessed on 4 December 2023) [44] for generating and evaluating the Ramachandran plot, while the ProSA-web server (https://prosa.services.came.sbg.ac.at/prosa.php, accessed on 7 December 2023) was employed to assess z-scores. Meanwhile, the ProSA-web’s z-score assesses the overall model quality by measuring the total energy deviation from that derived from random conformations, with values outside the typical range for native proteins indicating potential errors [45]. The Swiss-Model visually presents the distribution of phi (φ) and psi (ψ) dihedral angles, examining the protein structure.

### 2.8. Molecular Docking and MD Simulation

Molecular docking between the subunit vaccine construct as a ligand, with TLR2 and TLR4 as a receptor, was conducted using the ClusPro server (https://cluspro.bu.edu/, accessed on 19 December 2023) to understand the interaction between the receptor and ligand molecules. The ClusPro algorithm functions by systematically exploring the entire conformational space to identify low-energy docked structures. Subsequently, these structures undergo clustering, and the centers of the most substantial clusters are employed as probable models for the complex [46]. Docking was conducted for all vaccine constructs with the TLR2 and TLR4 models. The analysis of the docked models was performed using PyMOL v.2.5.4 [47] and LigPlot + v.2.2 [48] tools.

We conducted simulations specifically on the docked structures involving the consensus vaccine construct with TLR2 and TLR4, while for other variants, we used docked models to complement our research. Md simulation was performed using Gromacs version 2021.3 [49] using the “AMBER99SB-ILDN” force field. System neutralization involves the addition of sodium and chloride ions. Energy minimization was performed prior to simulation to configure a system with near-zero forces on each atom to achieve a local energy minimum using the steepest descent algorithm. NVT and NPT steps were performed for 1 ns each to maintain desired thermodynamic conditions throughout the simulation after equilibration. Following system equilibration, a 50 ns MD simulation was conducted for trajectory analysis.

## 3. Results

### 3.1. Epitope Prediction and Multi-Epitope Subunit Vaccine Construction

In this study, we focused on epitope prediction and the construction of a multi-epitope subunit vaccine. The development of the subunit vaccine included predicting various B-cell, CTL, and HTL epitopes, analyzing their properties, and ultimately, linking them together with linkers to create a multi-epitope vaccine. Utilizing the VP2 protein sequences from six distinct BTV serotypes (4, 10, 11, 17, 20, and 24), antigenic epitopes were predicted, subjected to property analysis, and then assembled to generate vaccine models.

The multiple sequence alignment of the VP2 protein sequences of all of the six serotypes showed a sequence identity of >69% (Supplementary Figure S1). We obtained a consensus sequence that integrates conserved regions to facilitate the development of a hexavalent subunit vaccine. The calculated antigenic propensity for all sequences exceeded one, signifying their substantial immunogenicity. Subsequently, these sequences were utilized for epitope prediction.

To predict B-cell epitopes for all of the seven sequences (six serotypes and one consensus sequence generated), four distinct servers, namely Ellipro, BcePred, SVMTrip, and ABCPred, were employed (Methods). The efficacy of vaccine-induced immune responses depends on chemical interactions, particularly with B-cell surface receptors. Vaccination in sheep causes dynamic changes in B-cell subpopulations, highlighting their crucial role in promoting an efficient immune response [50]. The VP2 protein modeling was conducted using the Robetta server, and the most Ramachandran-favored model was selected for input into Ellipro. All epitopes predicted by Ellipro were initially verified for the presence of the antigen sequence properties (hydrophilicity, flexibility, accessibility, turns, exposed surface, polarity, and antigenic propensity) using the IEDB server. Only those epitopes containing all specified properties within the predicted epitope region, with scores surpassing the average score for each property, were considered. Subsequently, epitopes were predicted using BcePred, and the regions highlighted in at least three physicochemical properties were selected. In SVMTrip, epitopes were predicted with default parameters, while in ABCPred, epitopes were predicted with threshold values set at 0.8. The epitopes, initially predicted by four different servers, underwent a comprehensive comparison process. Each epitope was assessed for potential overlaps with all other predicted epitopes, regardless of the server of origin. If an epitope from one server exhibited an overlap of more than 75% with another epitope (the overlap percentage was determined based on the size of the smaller epitope), it was considered a match. The cumulative score for each epitope was then determined based on the number of times it demonstrated such overlaps across different servers. Only those epitopes scoring above zero in at least three out of the four servers were retained for further analysis, indicating a consensus among the predictions. The B-cell epitope predictions for all the sequences, along with their scores, are provided in Supplementary Table S1A–G. Further, the prediction of CTL and HTL epitopes was conducted using the NetMHCpan—4.1 and NetMHCIIpan—2.1 servers, respectively. The synergistic actions of CTLs and HTLs are fundamental for defense against infections and the maintenance of immune homeostasis. HTL cells primarily assist in orchestrating immune responses by secreting cytokines and providing help to other immune cells, while CTL cells are primarily involved in directly killing infected or abnormal cells [51]. A set of 9 bovine MHC I and 14 bovine MHC II alleles were identified through comparison with the corresponding ovine MHC I and MHC II alleles (Methods). The prediction of CTL and HTL epitopes involved a screening process, wherein peptides were ranked based on prediction scores, and only those exhibiting strong binding affinities for each allele were retained. In instances where no strong binding peptide was identified for a particular allele, the top five weak binders were selected (Supplementary Tables S2A–G and S3A–G). Further refinement involved filtering the epitopes based on their prediction across multiple alleles, with a criterion of at least three alleles for CTL and four or five (depending on the total number of common alleles; choosing four when the count was low, otherwise five) alleles for HTL. In all the sequences, on average 14 CTL epitopes and 102 HTL epitopes were predicted.

Before progressing with the development of the subunit vaccine, an assessment was conducted on the selected B-cell, CTL, and HTL epitopes, involving checks for antigenicity, allergenicity, and toxicity. Epitopes failing to meet any of these critical criteria were excluded from consideration. Additionally, we examined the epitopes to find and remove any duplication or repetition. If there was more than 30% overlap between epitopes, the overlapping epitopes were marked, and the smaller one was removed, keeping the larger one to ensure that no epitopic region was omitted. On average, six B-cell, three CTL, and six HTL epitopes were finalized for all the VP2 sequences (Supplementary Table S4).

**Figure 1:**
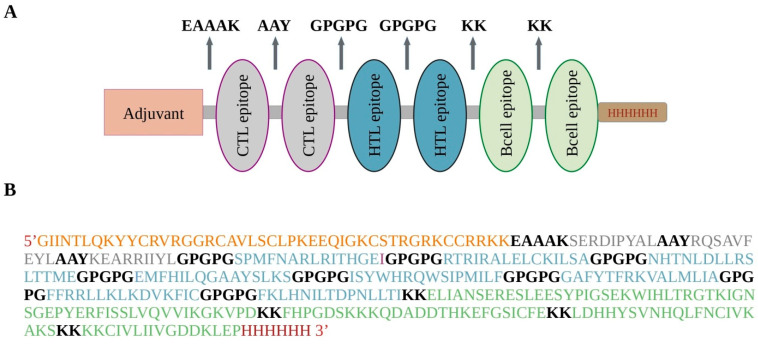
(**A**) Multi-epitope subunit vaccine structure. Components include the adjuvant (orange), cytotoxic T lymphocyte (CTL) epitopes (grey), helper-T lymphocyte (HTL) epitopes (teal), B-cell epitopes (green), and histidine tag (red). (**B**) Vaccine design of consensus sequence.

Subsequently, the design of the multi-epitope subunit vaccine unfolded, involving the integration of adjuvant, B-cell, CTL, and HTL epitopes. Beta defensin 3, functioning as a TLR2 and TLR4 agonist, served as the adjuvant. To interconnect the chosen epitopes and enhance the induction of immune responses, linkers were incorporated into the vaccine construction. The EAAAK linker was introduced to separate the adjuvant and CTL epitopes, while the KK linker was applied between B-cell epitopes. The AAY and GPGPG linkers were employed to segregate the CTL and HTL epitopes, respectively. Linkers were applied to join epitopes across all sequences (Figure 1A), encompassing six serotypes and a consensus. The overall length of the final subunit vaccine of the consensus sequence was 374 amino acids, which contained one EAAAK linker, eight GPGPG linkers, two AAY linkers, and four KK linkers (Figure 1B). The vaccine length and the respective number of linkers used to join the vaccine constructs of the six serotypes are given in Supplementary Table S5A.

### 3.2. Modeling and Structural Refinement of Subunit Vaccine Constructs

Before delving into the modeling and structural refinement of the subunit vaccine constructs, the antigenicity, and allergenicity of the final vaccine designs were evaluated. Employing Vaxijen 2.0 and AllerTOP 2.0 servers, the assessments revealed promising results, indicating that all vaccine constructs, spanning the consensus and serotypes 4, 10, 11, 17, 20, and 24, were non-allergenic and antigenic. The Vaxijen scores, ranging from 0.5672 to 0.7297, underscored their potential as effective antigens. With the confirmation of their ability to elicit an immune response, the next step involved a thorough examination of their physicochemical properties using the ProtParam tool. We examined the molecular weight, isoelectric point (PI), aliphatic index, and GRAVY (grand average of hydropathy) (Supplementary Table S5B), unveiling insights into the stability and characteristics of the vaccine constructs. Notably, all the constructs exhibited a PI greater than nine, indicating their basic nature. The aliphatic index, representing the volume occupied by aliphatic side chains (alanine, valine, isoleucine, and leucine), serves as a positive factor for enhancing the thermostability of globular proteins [52]. Additionally, GRAVY reflects the average hydropathy value of a protein, with positive values indicating hydrophobicity and negative values indicating hydrophilicity [53]. All vaccine constructs displayed a high aliphatic index, suggesting thermostability, and negative GRAVY values, indicating hydrophilicity. Furthermore, the instability index, a predictor of protein stability, was below 40 for all the constructs, signifying their stable nature.

Advancing from these physiochemical evaluations, our analysis delved into the secondary and tertiary structures of the final vaccine constructs. The secondary structure prediction for the final vaccine constructs was conducted using the online server PSIPRED. The corresponding percentage of amino acids involved in coil, β-strand, and α-helix formation in all the vaccine constructs is given in Supplementary Table S5C. For tertiary structure prediction of the multi-epitope vaccine constructs, the Robetta server was used. This server dissects input sequences into potential domains, generating models for domains with sequence homology to known protein structures through comparative modeling. For domains lacking homology, it utilizes the Rosetta de novo structure prediction method. The server took an amino acid sequence as the input data and produced five tertiary models as the output. To determine the most suitable model for the vaccine constructs, a selection was made based on a low Angstroms error estimate for the overall sequence length.

**Figure 2:**
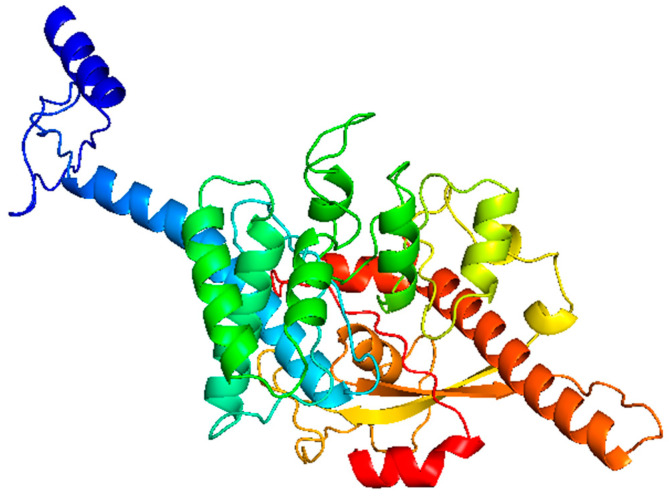
Tertiary structure of subunit vaccine of consensus sequence.

**Figure 3:**
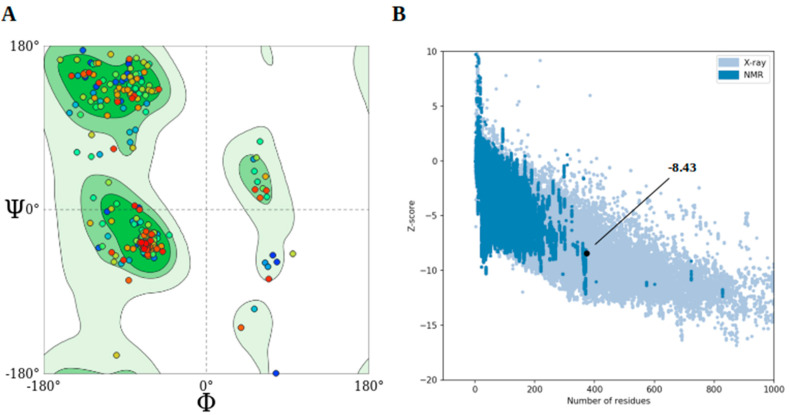
(**A**) Ramachandran plot with 94.9% residues in favored region and (**B**) ProSA-server Z-score, of the consensus vaccine refined model.

The tertiary (3D) structure of the final vaccine construct underwent additional refinement using the GalaxyRefine server. To evaluate the refined models’ quality, we utilized the Swiss-Model structure assessment server and the ProSA-web server. The Z-scores of all the refined models are mentioned in Supplementary Table S5B. GalaxyRefine generated five models after refinement, out of which the best model was selected based on the lowest MolProbity score coupled with the presence of the most Ramachandran-favored residues. The tertiary model of the consensus vaccine construct is shown in Figure 2. The galaxy refines results for all the vaccine constructs are provided in Supplementary Table S6. For the consensus sequence, the refined model showed a 94.89% favored region in the Ramachandran plot, which is the best score among all the refined models, and a z-score of −8.43, indicating its overall good quality. Furthermore, the assessment metrics for the consensus refined model included a GDT-HA value of 0.9666, RMSD of 0.367, MolProbity score of 2.056, clash score of 15.9, and poor rotamers of 0.3. Validation of the refined models of consensus (Figure 3A,B) and the other six constructs (Supplementary Figure S2A–G) suggested that they had good quality and stability and were further subjected to docking analysis.

### 3.3. Molecular Docking and MD Simulation

**Figure 4:**
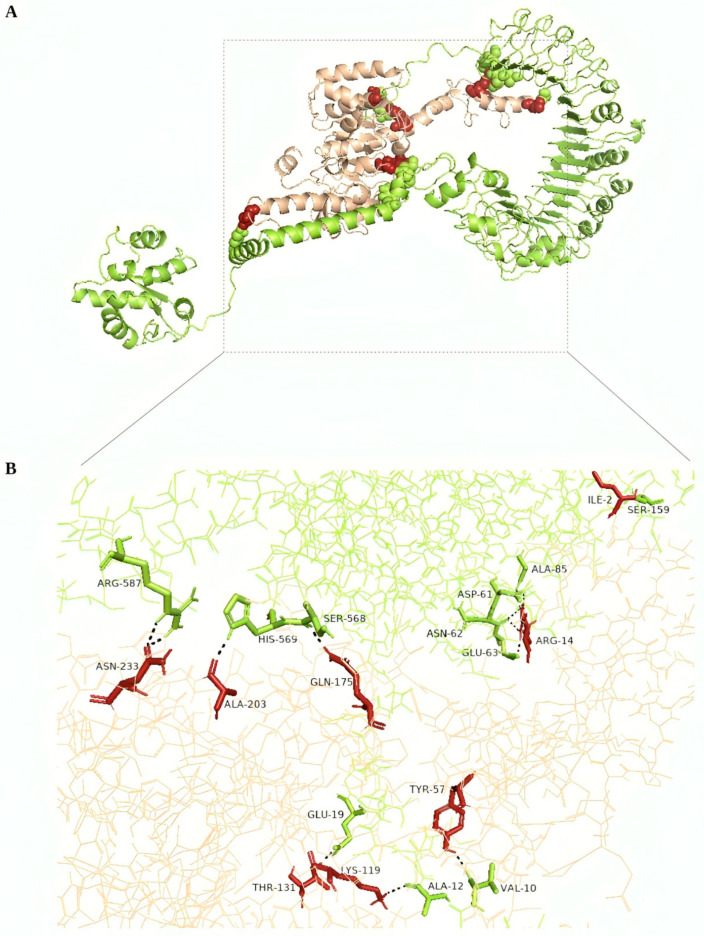
(**A**) Visualization of docked TLR2—consensus vaccine complex. (**B**) Enlarged view highlighting the interacting residues in TLR2 (green) and consensus vaccine (brown). Hydrogen bonds are shown as the black dashed line.

**Figure 5:**
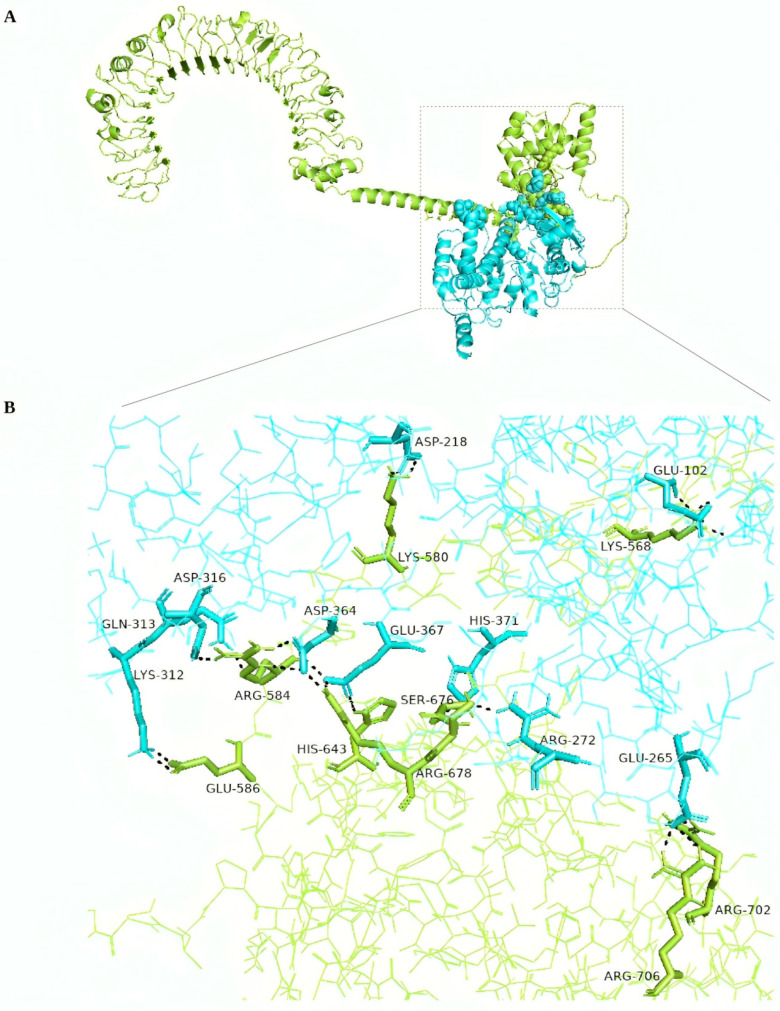
(**A**) Visualization of docked TLR4—consensus vaccine complex. (**B**) Enlarged view highlighting the interacting residues in TLR4 (green) and consensus vaccine (blue). Hydrogen bonds are shown as the black dashed line.

**Figure 6:**
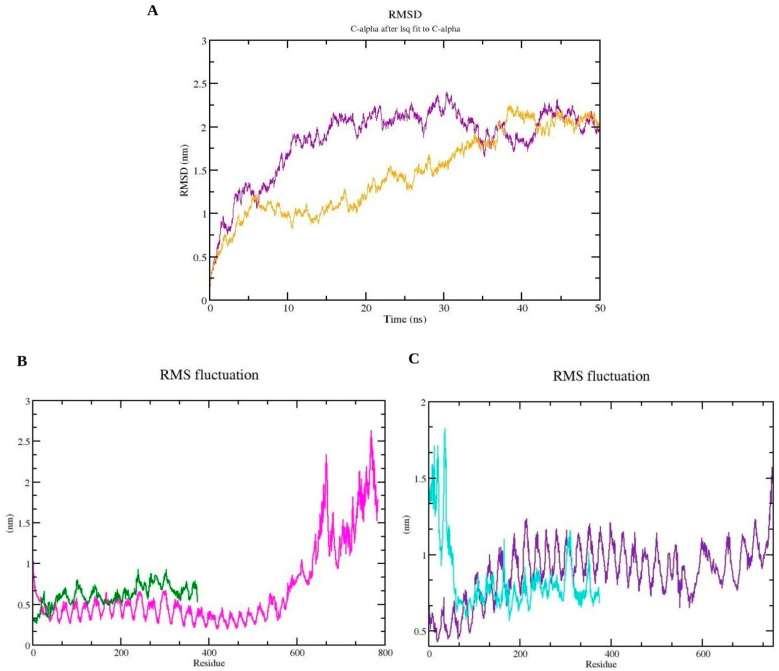
MD simulation results of consensus vaccine complex with TLR2 and TLR4 over 50 ns trajectory. (**A**) RMSD graph of TLR2-vaccine (purple) and TLR4-vaccine (yellow) complex. (**B**) RMSF graph of the TLR2 protein (magenta) and vaccine construct (green). (**C**) RMSF graph of the TLR4 protein (violet) and vaccine construct (cyan).

Having successfully refined the models, the next crucial step was to evaluate their interaction with immune receptors TLR2 and TLR4 through molecular docking. To evaluate the interaction between the refined models and immune receptors TLR2 and TLR4, molecular docking was conducted using the online server Cuspro 2.0, yielding 30 models as the output. Cluspro recommends ranking models based on cluster size (which is how the models are ranked coming out of Cluspro), and thus, the first docked model was chosen in each case. The resulting docked complex of the consensus vaccine construct with both TLR2 and TLR4, along with an overview of their interacting residues, is illustrated in Figure 4 and Figure 5. Detailed information on interacting residues for all docked complexes is provided in Supplementary Figure S3A–G. The docked complexes of the consensus sequence were further employed for MD simulation studies, which were performed using Gromacs 2021.3 software between the vaccine molecule with TLR2 and TLR4 for 50 ns. MD simulations are crucial, as they provide detailed insights into atomic behavior, guiding functional understanding, and assisting in the design of molecules. By predicting atomic movements and responses, MD simulations complement experiments, offering high-resolution insights into conformational changes, ligand binding, and protein folding [54]. The calculation of root-mean-square deviation (RMSD) for both of the docked vaccine complexes was performed and the average RMSD values for the vaccine-TLR2 and vaccine-TLR4 complexes were 1.84907 Å and 1.45717 Å, respectively (Figure 6A), underscoring the structural stability during the interaction. Additionally, an assessment of protein flexibility across amino acid residues was conducted using the root-mean-square fluctuation (RMSF) score (Figure 6B,C). The RMSF profile of both the vaccine and the receptors reveals that the majority of amino acid residues in the complexes exhibit an RMSF profile below 2.5 Å. The simulation analysis indicates the formation of a stable complex by the multi-epitope vaccine protein.

## 4. Discussion

While bluetongue disease is prevalent in sheep and certain wildlife, factors influencing disease expression vary. Ruminants exhibit antiviral responses, including antibodies against the outer capsid protein VP2, which neutralizes the virus. Diagnostic serologic tests rely on antibodies targeting common epitopes in the bluetongue serogroup [55]. BTV vaccination is essential globally due to limited options. Inactivated vaccines provide serotype-specific protection, while modified live vaccines raise safety concerns. Current vaccines cannot differentiate infected from vaccinated animals (DIVA), emphasizing the need for effective strategies to control and prevent bluetongue outbreaks, which is crucial for economic stability [56]. In this study, we employed immunoinformatics to design a multi-epitope subunit vaccine utilizing the VP2 capsid protein of *Ovis aries*. The focus was on targeting six BTV serotypes: 4, 10, 11, 17, 20, and 24, as well as a consensus sequence derived from these six serotypes. Our vaccine contains a suitable adjuvant (beta defensin 3), HTL, CTL, and B-cell epitopes that are joined by suitable linkers. In vaccine development, HTL supports the induction of both cellular and humoral immune responses. The effective optimization of the HTL function stands as a critical factor in the creation of vaccines with heightened efficacy [57]. The contribution of CD8+ CTLs is important, as it plays a vital role in eliminating intracellular pathogens and tumors, forming long-lasting memory cells for rapid future responses. Their importance is particular in vaccine efficacy, especially for addressing pathogens that are resistant to neutralization [58]. Lastly, recognizing the significance of B cells in providing humoral immunity in mammals [25], our vaccine strategically incorporates B-cell epitopes, HTL, and CTL for a comprehensive immune response. The inclusion of a consensus sequence in our multi-epitope subunit vaccine is of significance due to its potential to address the antigenic variability observed among BTV serotypes.

In developing our subunit vaccine, we utilized a comprehensive epitope-prediction approach. B-cell epitopes were predicted through Ellipro, SVMTriP, ABCPred, and BcePred servers. T-cell epitope prediction for sheep involved selecting bovine alleles with >90% coverage and identity with ovine MHC alleles. To tailor T-cell epitope prediction for sheep, bovine alleles with >90% coverage and identity with ovine MHC alleles were carefully selected. Subsequently, HTL and CTL epitopes were predicted using these alleles, focusing on strong binding affinity. The epitopes underwent screening for toxicity, antigenicity, and allergenicity, ensuring the final subunit vaccine constructs are thermostable, antigenic, non-toxic, and non-allergenic. Through molecular docking, we obtained valuable insights into the intermolecular interactions and dynamics of all the vaccine proteins with TLR2 and TLR4 receptors. An MD simulation was conducted for the consensus sequence vaccine construct, and the resulting 50 ns RMSD trajectory demonstrates a consistent and stable interaction within the vaccine–receptor complex.

Addressing the urgency and severity of BTV outbreaks, computational predictions play a crucial role in guiding vaccine design. Traditional vaccine creation for BTV is a time-intensive process with high failure rates. However, optimized subunit vaccines, specifically targeting antigenic portions, offer a faster route for testing and release. These subunit vaccines, devoid of live pathogens, mitigate the risk of pathogenicity reversal, making them suitable for immune-suppressed animals while eliciting better immunity. Computational studies indicate the potential efficacy and safety of our multi-epitope subunit vaccine against BTV, emphasizing the need for synthesis and experimental evaluation to ascertain its immunogenic potency.

## Data Availability

All original contributions from this study are included in the article/Supplementary Materials.

## Supplementary Materials

The following supporting information can be downloaded at https://www.mdpi.com/article/10.3390/pathogens13110944/s1: Supplementary Figure S1. Identity matrix of the Multiple Sequence alignment of VP2 proteins from the six distinct serotypes (4, 10, 11, 17, 20, and 24) of BTV. Supplementary Figure S2. Ramachandran plots and Z score validation for the structural conformations of refined tertiary vaccine structures of serotypes 4 (A, B), 10 (C, D), 11 (E, F), 17 (G, H), 20 (I, J), and 24 (K, L). Supplementary Figure S3. Visualization of the amino acid interaction between TLR2 (chain B) and vaccine constructs of serotypes 4 (A), 10 (B), 11 (C), 17 (D), 20 (E), 24 (F), and consensus (G) (chain A). Supplementary Figure S4. visualization of the amino acid interaction between TLR4 (chain B) and vaccine constructs of serotypes 4 (A), 10 (B), 11 (C), 17 (D), 20 (E), 24 (F), and consensus (G) (chain A). Supplementary Table S1. (A–G) B-cell epitope predictions for all sequences, including their scores. Supplementary Tables S2. (A–G) Prediction of CTL epitopes, with peptides ranked based on prediction scores. Supplementary Tables S3. (A–G) Prediction of HTL epitopes, with peptides ranked based on prediction scores. Supplementary Table S4. Final B-cell, CTL, and HTL epitopes for all serotypes used in the vaccine design. Supplementary Table S5. (A) Vaccine length and the number of linkers used to join the vaccine constructs for the six serotypes. (B) Molecular weight, isoelectric point (PI), aliphatic index, and GRAVY (grand average of hydropathy) of all the vaccine constructs. (C) Percentage of amino acids involved in coil, β-strand, and α-helix formation for all vaccine constructs. Supplementary Table S6. GalaxyRefine results for all vaccine constructs, including five refined models where the best model was selected based on the lowest MolProbity score and the highest number of Ramachandran-favored residues.
