# Supplementary material for: In Silico Development of a Multi-Epitope Subunit Vaccine against Bluetongue Virus in Ovis aries Using Immunoinformatics
Source: Pathogens. 2024 Oct 29;13(11):944. doi: 10.3390/pathogens13110944 (PMC11597718; doi:10.3390/pathogens13110944)
Supplement: Supplementary file 1 [file pathogens-13-00944-s001.zip › Supplementary figures.pdf]

Percent Identity Matrix - created by Clustal2.1

Figure S1

1: tr|A0PCN7|A0PCN7_BTV1X	100.00	74.35	70.82	70.79	73.33	70.29
2: tr|A0PCQ1|A0PCQ1_9VIRU	74.35	100.00	71.10	69.92	72.36	70.47
3: tr|A0PCN8|A0PCN8_9VIRU	70.82	71.10	100.00	73.72	71.65	71.13
4: tr|A0PCP4|A0PCP4_9VIRU	70.79	69.92	73.72	100.00	74.87	72.77
5: tr|Q2VEZ8|Q2VEZ8_9VIRU	73.33	72.36	71.65	74.87	100.00	77.62
6: tr|A0PCP7|A0PCP7_9VIRU	70.29	70.47	71.13	72.77	77.62	100.00

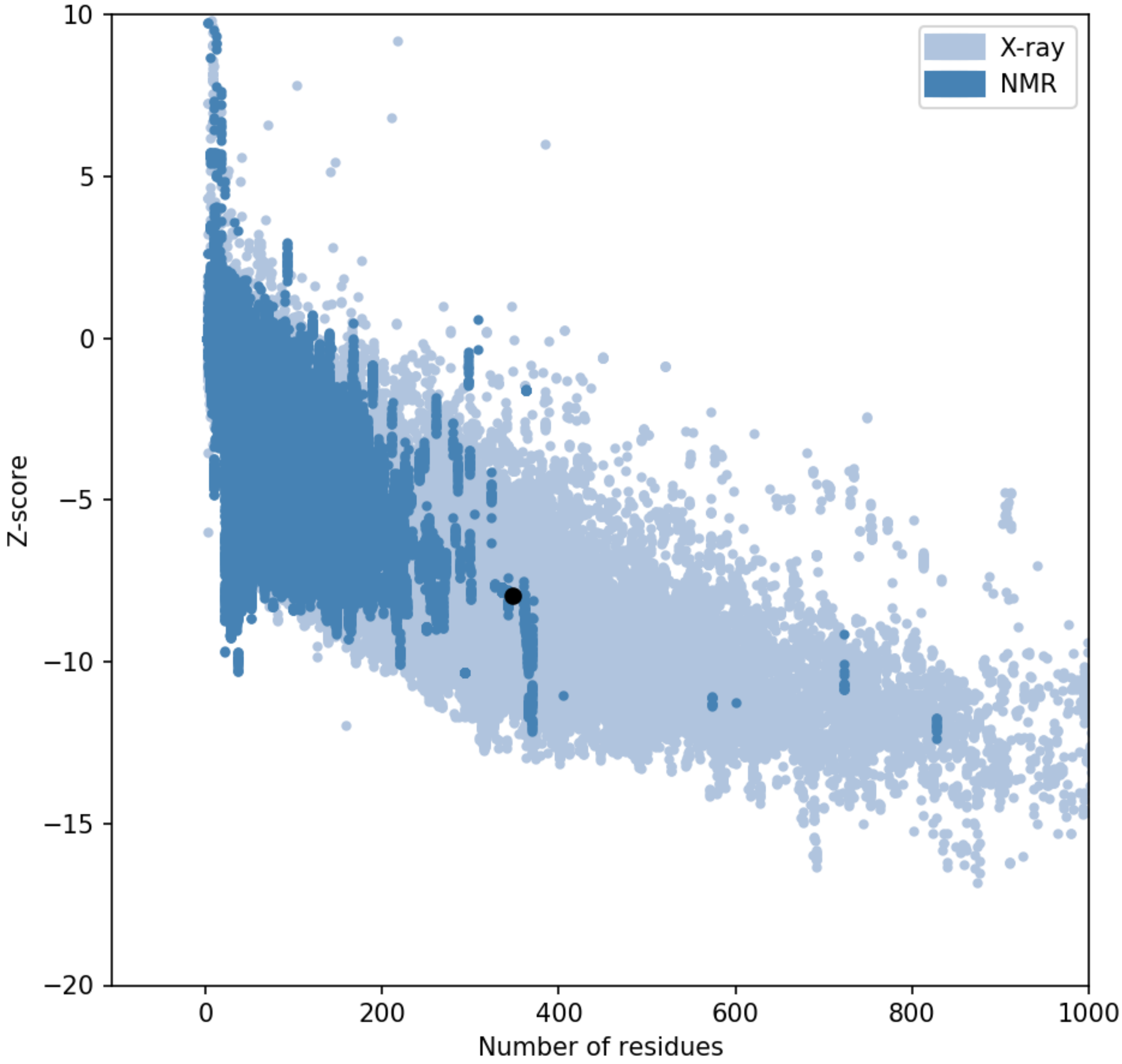
X-ray
NMR
Z-score
Number of residues
10
5
0
−5
−10
−15
−20
0
200
400
600
800
1000

A

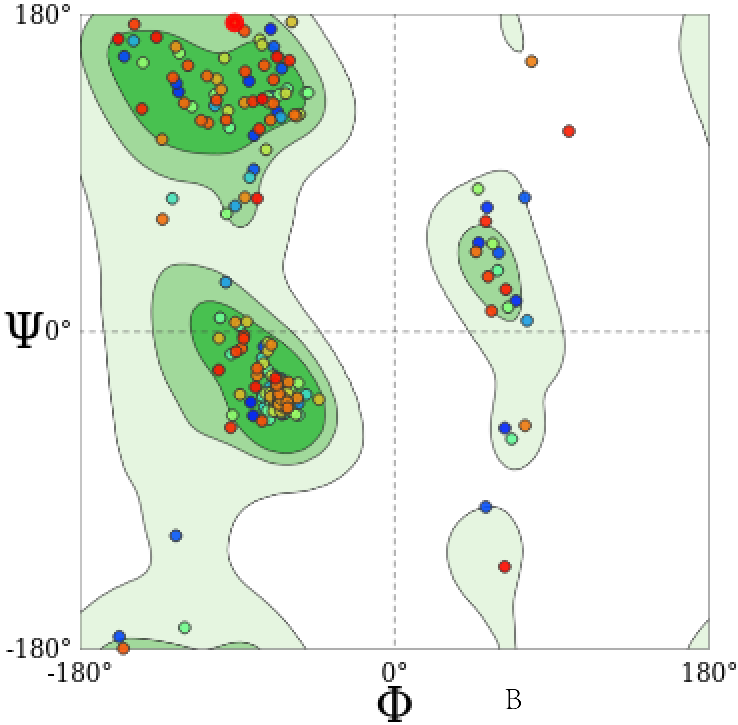

B

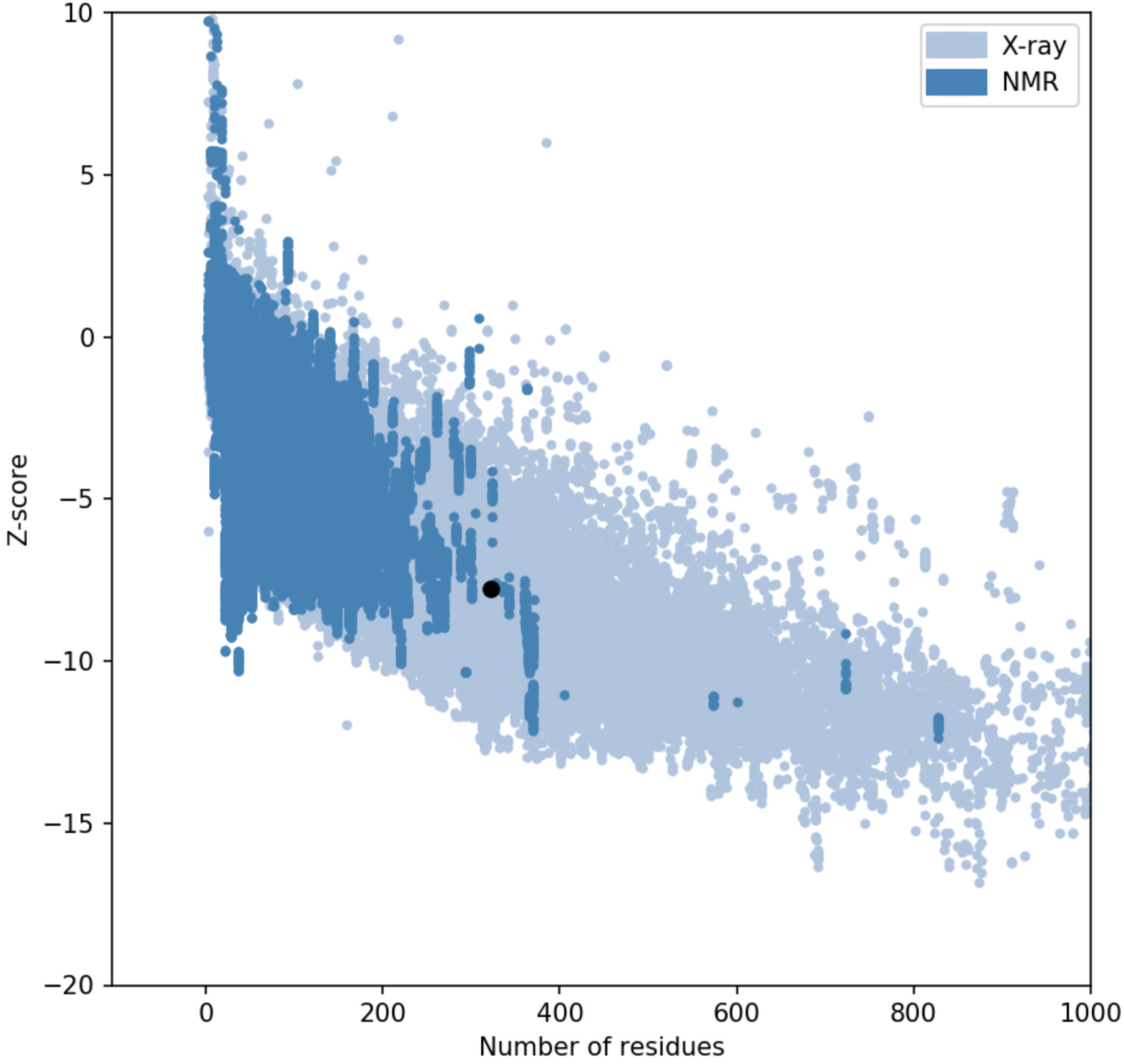
X-ray
NMR
Z-score
Number of residues
10
5
0
−5
−10
−15
−20
0
200
400
600
800
1000
C

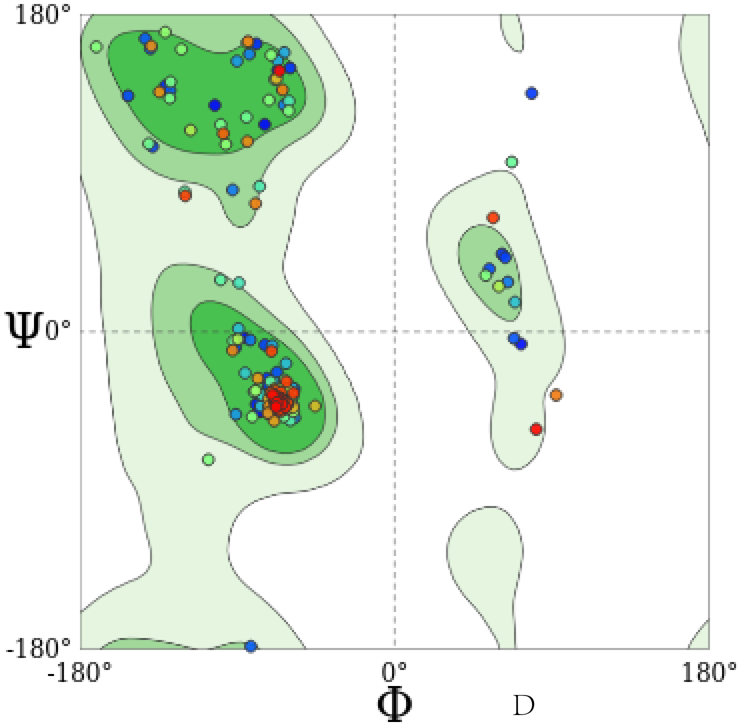

D

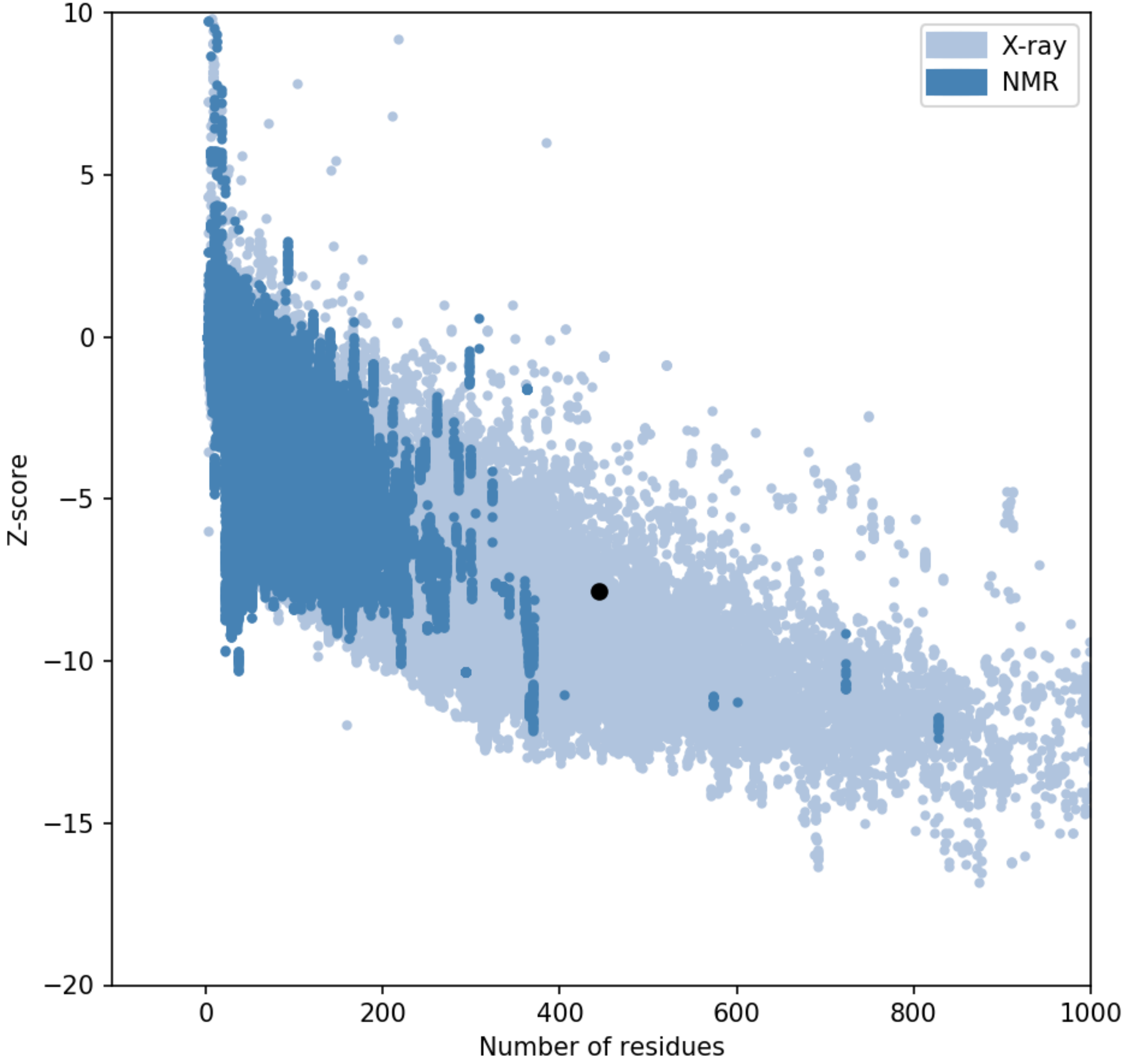
X-ray
NMR
Z-score
Number of residues
10
5
0
−5
−10
−15
−20
0
200
400
600
800
1000

E

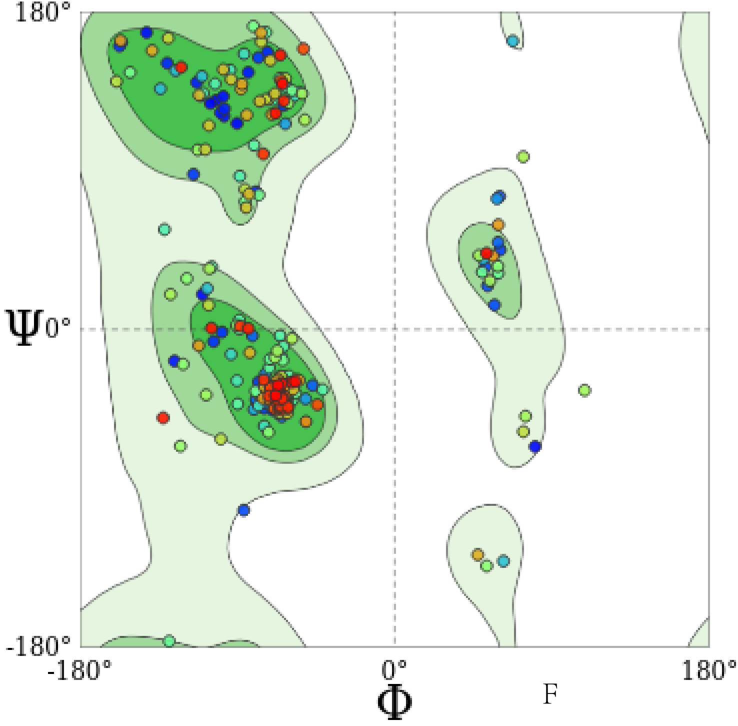
180°
Ψ0°
-180°
-180°
0°
180°
Φ
F

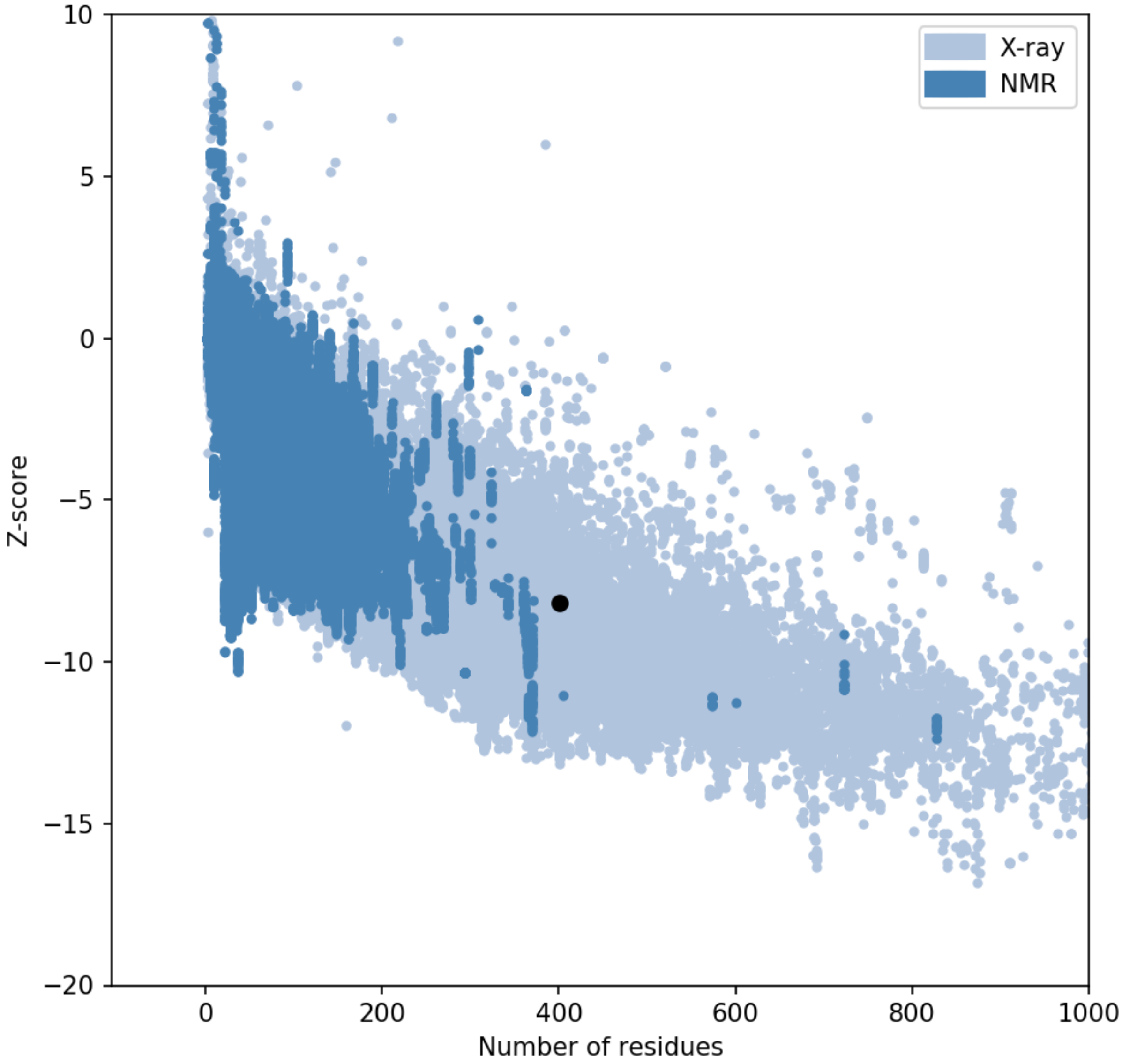
10
5
0
−5
−10
−15
−20
Z-score
0
200
400
600
800
1000
Number of residues
X-ray
NMR

G

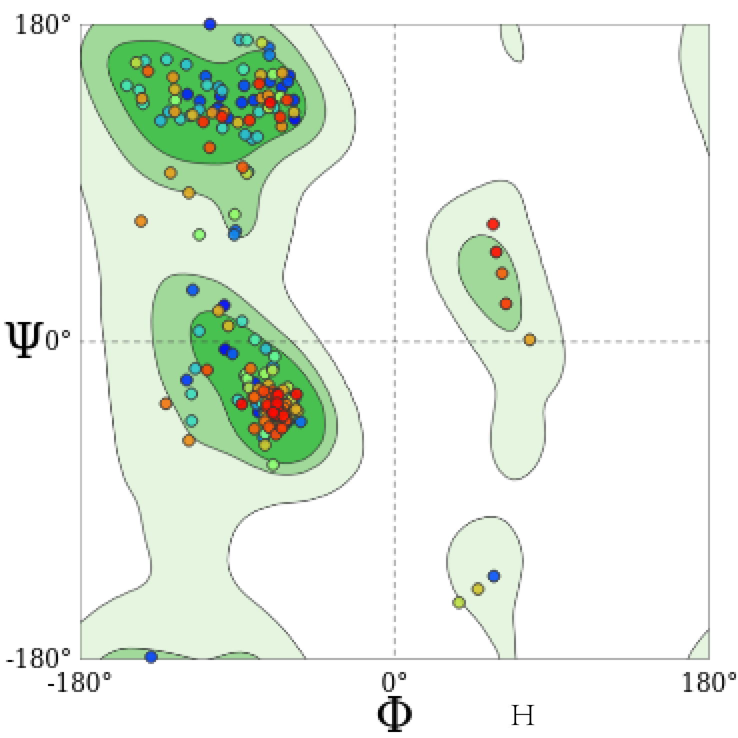
180°
Ψ 0°
-180°
-180°
0°
180°
Φ
H

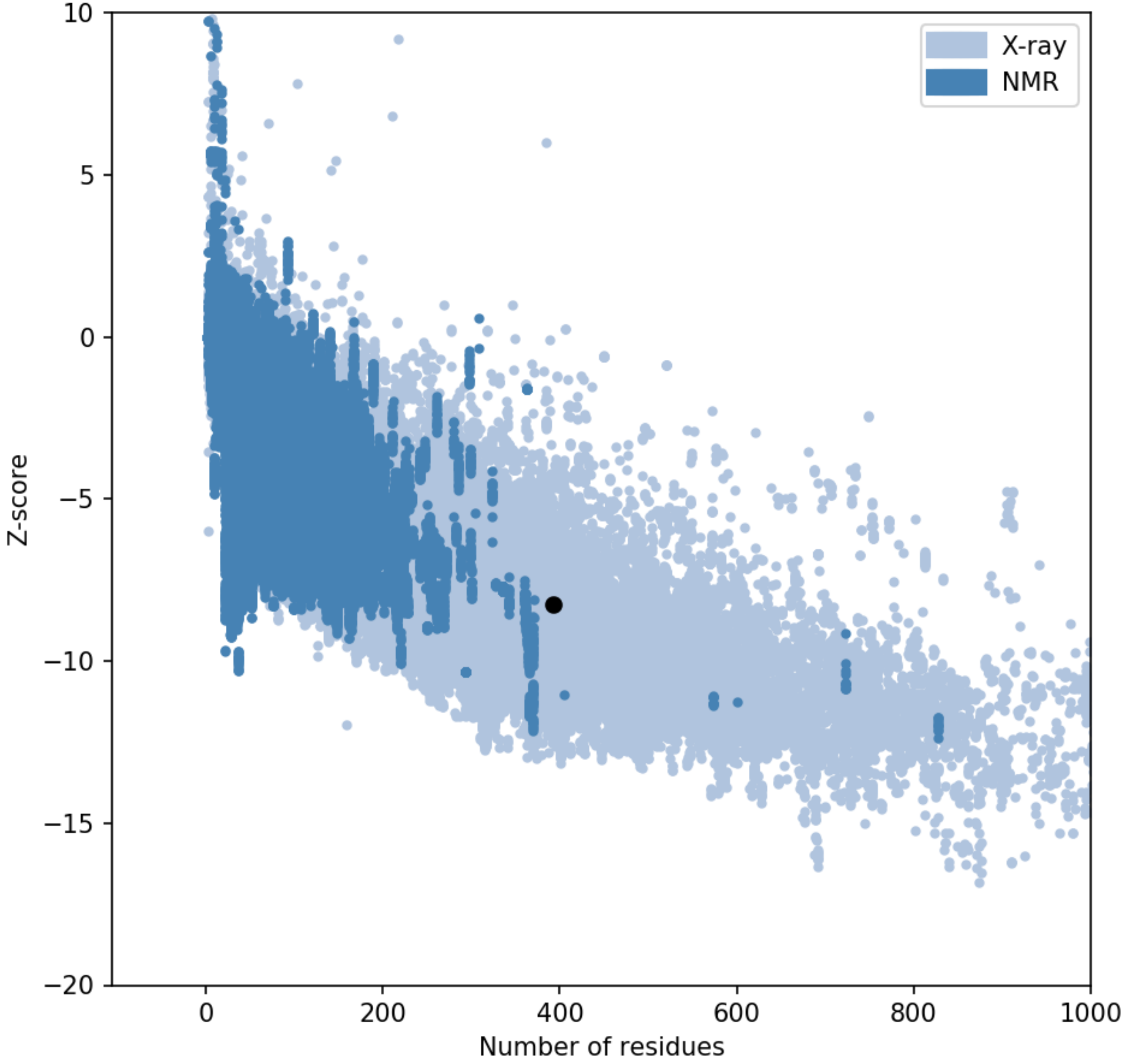

X-ray
NMR
Z-score
Number of residues
10
5
0
−5
−10
−15
−20
0
200
400
600
800
1000

I

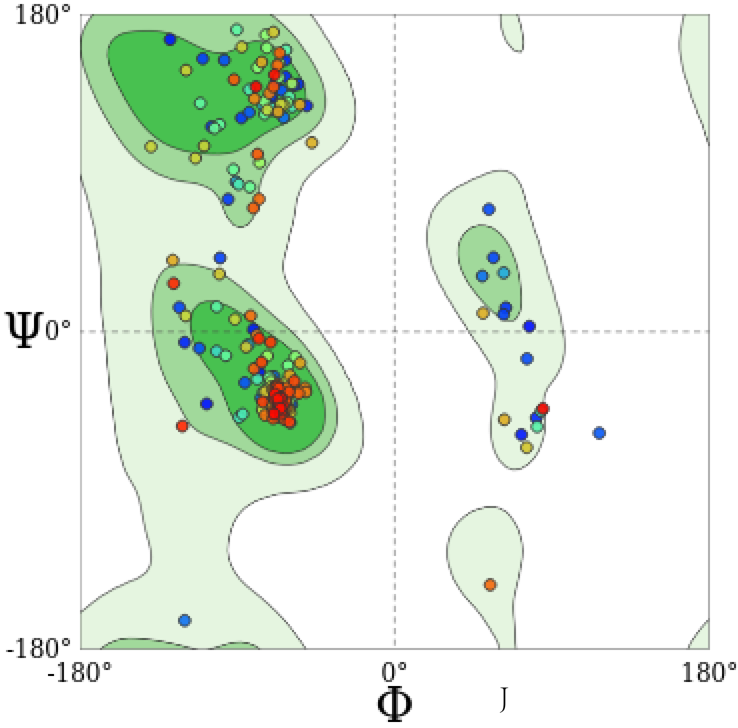

J

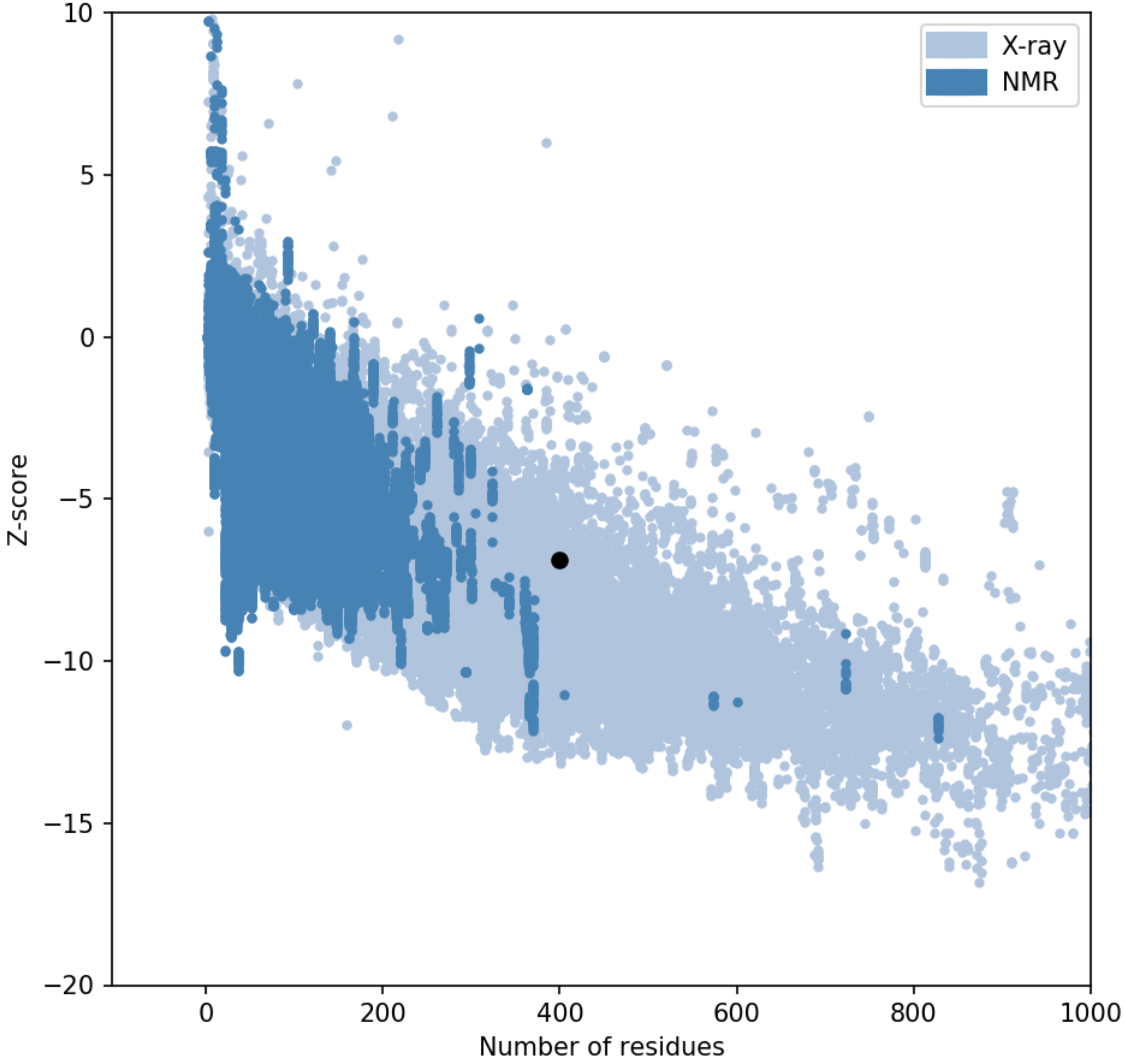
X-ray
NMR
Z-score
Number of residues
10
5
0
−5
−10
−15
−20
0
200
400
600
800
1000

K

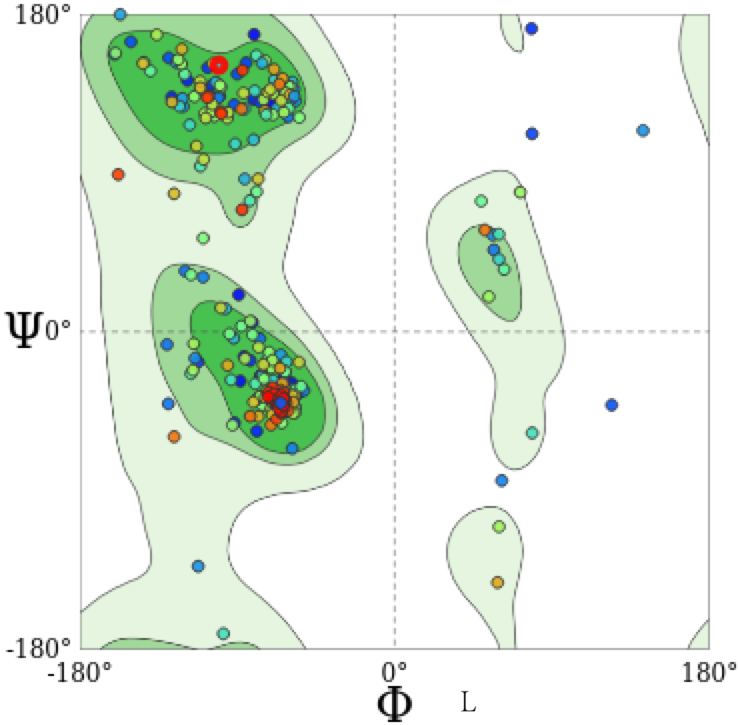
180°
Ψ 0°
-180°
-180°
0°
180°
Φ
L

Figure S3

A

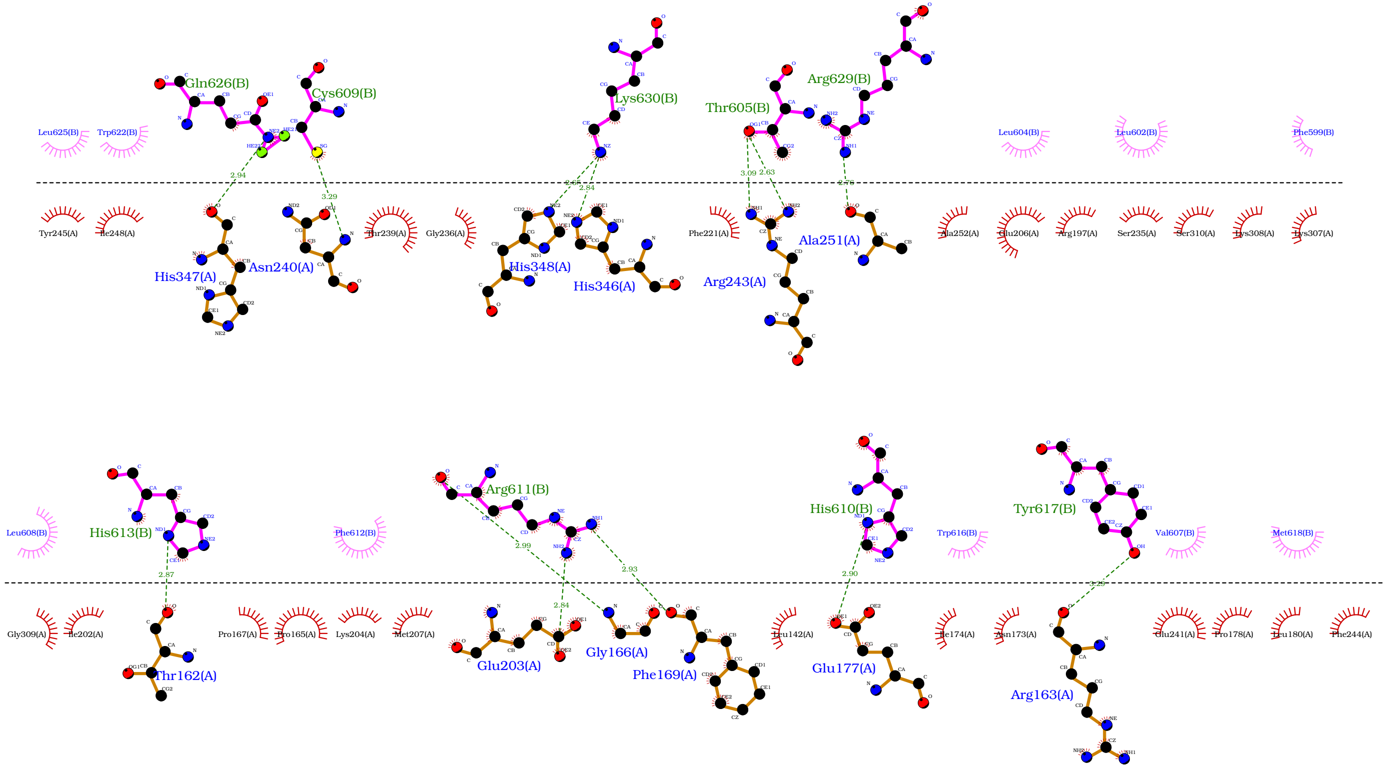
Gln626(B)
Cys609(B)
Lys630(B)
Thr605(B)
Arg629(B)
Leu625(B)
Trp622(B)
Leu604(B)
Leu602(B)
Phe599(B)
2.94
3.29
2.84
3.09
2.63
2.76
Tyr245(A)
Ile248(A)
Thr239(A)
Gly236(A)
Phe221(A)
Ala251(A)
Ala252(A)
Glu206(A)
Arg197(A)
Ser235(A)
Ser310(A)
Lys308(A)
Lys307(A)
His347(A)
Asn240(A)
His348(A)
His346(A)
Arg243(A)
Arg611(B)
His610(B)
Tyr617(B)
Leu608(B)
His613(B)
Phe612(B)
Trp616(B)
Val607(B)
Met618(B)
2.87
2.99
2.93
2.84
2.90
Gly309(A)
Ile202(A)
Pro167(A)
Pro165(A)
Lys204(A)
Met207(A)
Leu142(A)
Ile174(A)
Asn173(A)
Glu241(A)
Pro178(A)
Leu180(A)
Phe244(A)
Thr162(A)
Glu203(A)
Gly166(A)
Phe169(A)
Glu177(A)
Arg163(A)

B

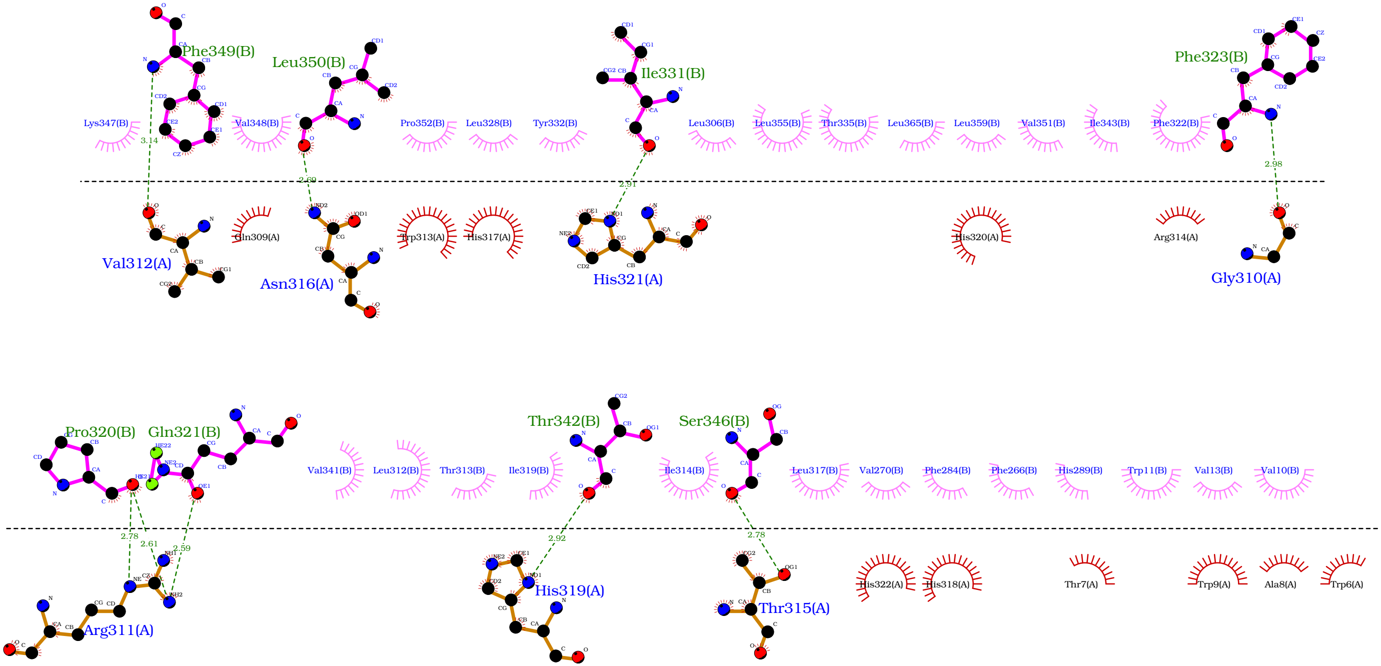
Phe349(B)
Leu350(B)
Ile331(B)
Phe323(B)
Lys347(B)
Val348(B)
Pro352(B)
Leu328(B)
Tyr332(B)
Leu306(B)
Leu355(B)
Thr335(B)
Leu365(B)
Leu359(B)
Val351(B)
Ile343(B)
Phe322(B)
3.14
2.69
2.91
2.98
Gln309(A)
Trp313(A)
His317(A)
His320(A)
Arg314(A)
Val312(A)
Asn316(A)
His321(A)
Gly310(A)
Pro320(B)
Gln321(B)
Thr342(B)
Ser346(B)
Val341(B)
Leu312(B)
Thr313(B)
Ile319(B)
Ile314(B)
Leu317(B)
Val270(B)
Phe284(B)
Phe266(B)
His289(B)
Trp11(B)
Val13(B)
Val10(B)
2.78
2.61
2.59
2.92
2.78
His322(A)
His318(A)
Thr7(A)
Trp9(A)
Ala8(A)
Trp6(A)
Arg311(A)
His319(A)
Thr315(A)

C

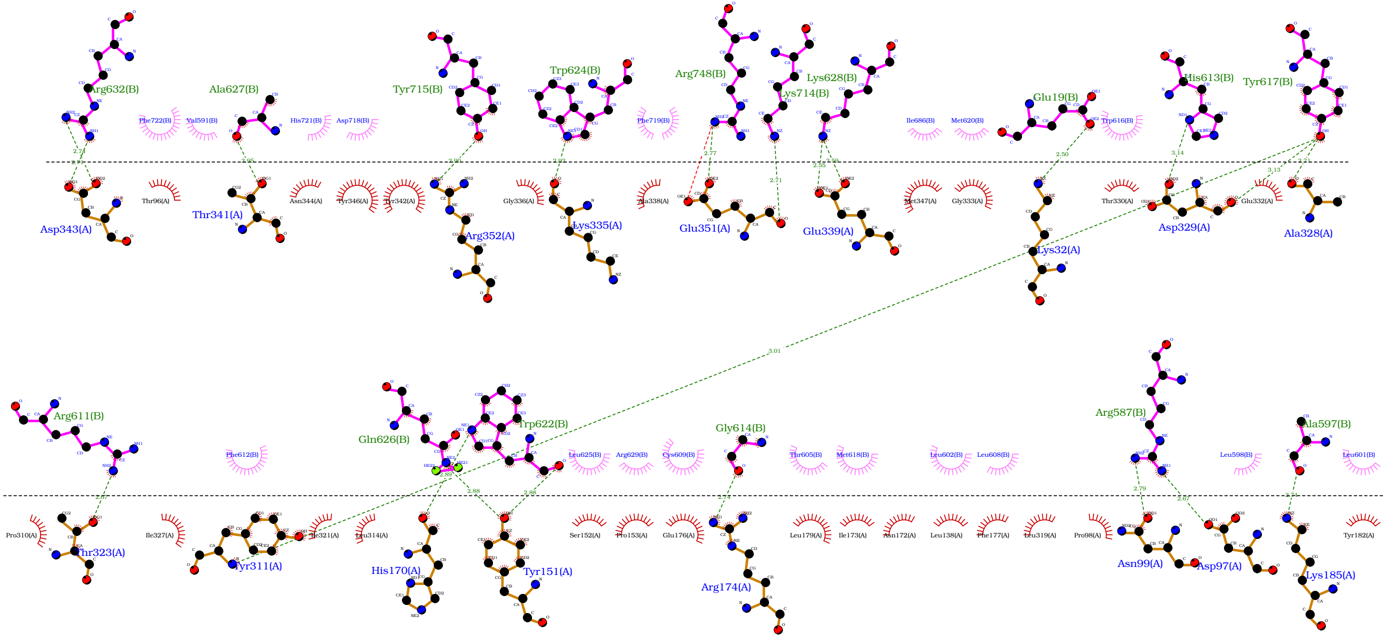

Arg632(B)
Ala627(B)
Tyr715(B)
Trp624(B)
Arg748(B)
Lys628(B)
Lys714(B)
Glu19(B)
His613(B)
Tyr617(B)
Asp343(A)
Thr341(A)
Arg352(A)
Lys335(A)
Glu351(A)
Glu339(A)
Lys32(A)
Asp329(A)
Ala328(A)
Arg611(B)
Gln626(B)
Trp622(B)
Gly614(B)
Arg587(B)
Ala597(B)
Thr323(A)
Tyr311(A)
His170(A)
Tyr151(A)
Arg174(A)
Asn99(A)
Asp97(A)
Lys185(A)

D

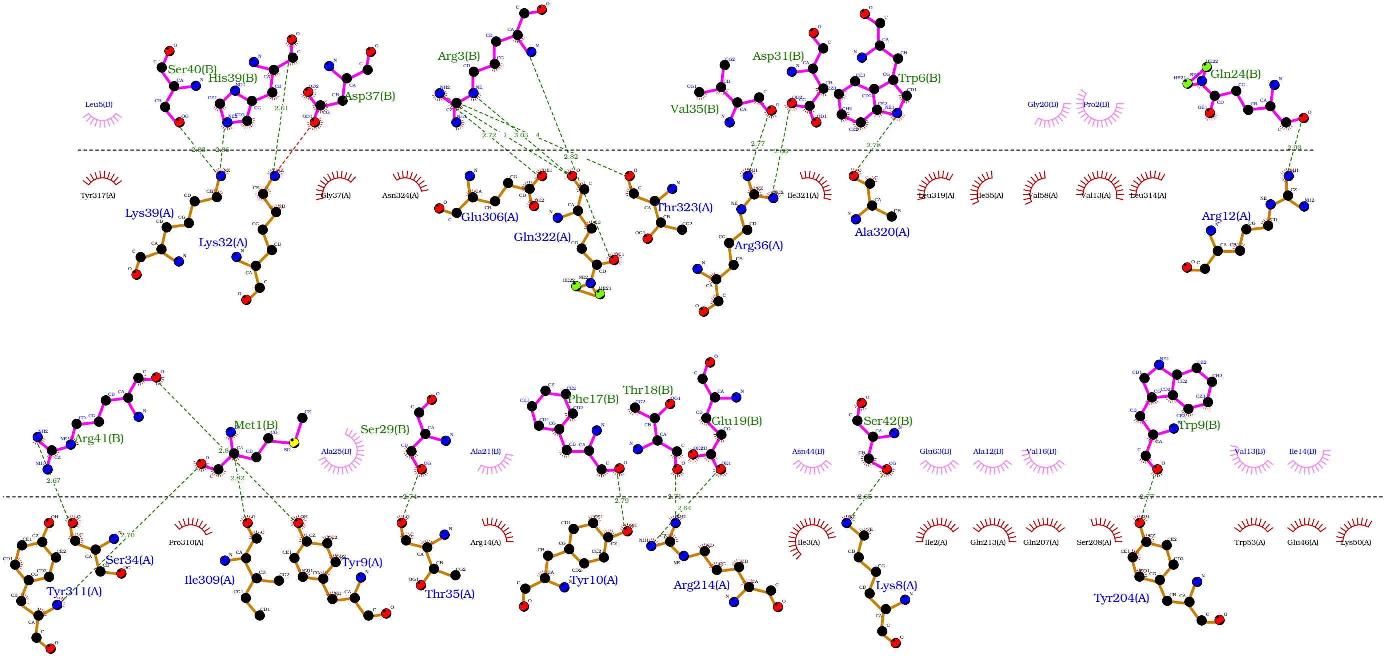
Ser40(B)
His39(B)
Asp37(B)
Arg3(B)
Asp31(B)
Trp6(B)
Gln24(B)
Val35(B)
Leu5(B)
Gly20(B)
Pro2(B)
Tyr317(A)
Lys39(A)
Lys32(A)
Gly37(A)
Asn324(A)
Glu306(A)
Gln322(A)
Thr323(A)
Arg36(A)
Ile321(A)
Ala320(A)
Leu319(A)
Ile55(A)
Val58(A)
Val13(A)
Leu314(A)
Arg12(A)
Arg41(B)
Met1(B)
Ser29(B)
Phe17(B)
Thr18(B)
Glu19(B)
Ser42(B)
Trp9(B)
Ala25(B)
Ala21(B)
Asn44(B)
Glu63(B)
Ala12(B)
Val16(B)
Val13(B)
Ile14(B)
Ser34(A)
Tyr311(A)
Pro310(A)
Ile309(A)
Tyr9(A)
Arg14(A)
Thr35(A)
Tyr10(A)
Arg214(A)
Ile3(A)
Lys8(A)
Ile2(A)
Gln213(A)
Gln207(A)
Ser208(A)
Tyr204(A)
Trp53(A)
Glu46(A)
Lys50(A)

E

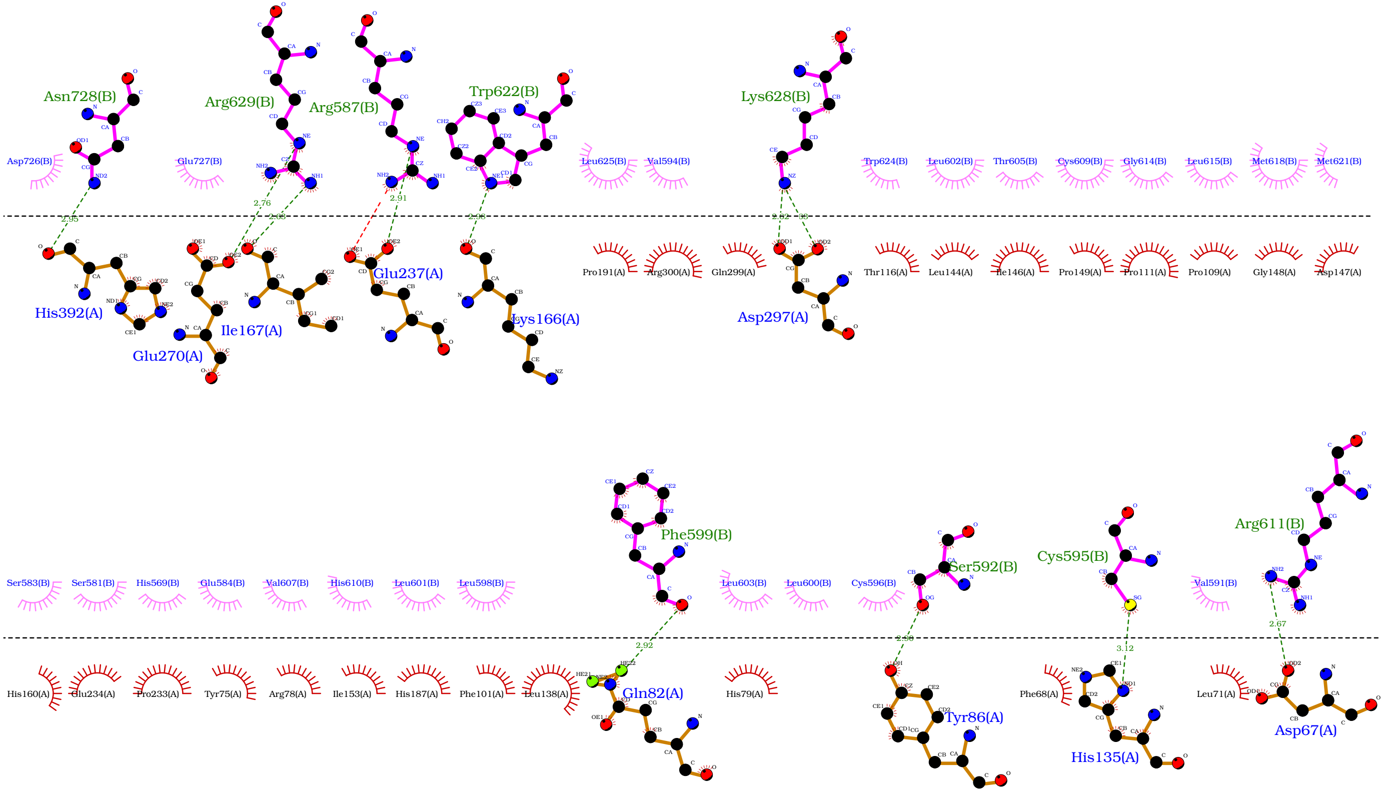

Asn728(B)
Arg629(B)
Arg587(B)
Trp622(B)
Lys628(B)
Asp726(B)
Glu727(B)
Leu625(B)
Val594(B)
Trp624(B)
Leu602(B)
Thr605(B)
Cys609(B)
Gly614(B)
Leu615(B)
Met618(B)
Met621(B)
2.95
2.76
2.68
2.91
2.98
2.82
Pro191(A)
Arg300(A)
Gln299(A)
Thr116(A)
Leu144(A)
Ile146(A)
Pro149(A)
Pro111(A)
Pro109(A)
Gly148(A)
Asp147(A)
His392(A)
Ile167(A)
Glu270(A)
Glu237(A)
Lys166(A)
Asp297(A)
Phe599(B)
Ser592(B)
Cys595(B)
Arg611(B)
Ser583(B)
Ser581(B)
His569(B)
Glu584(B)
Val607(B)
His610(B)
Leu601(B)
Leu598(B)
Leu603(B)
Leu600(B)
Cys596(B)
Val591(B)
2.92
3.12
2.67
His160(A)
Glu234(A)
Pro233(A)
Tyr75(A)
Arg78(A)
Ile153(A)
His187(A)
Phe101(A)
Leu138(A)
Gln82(A)
His79(A)
Tyr86(A)
Phe68(A)
His135(A)
Leu71(A)
Asp67(A)

F

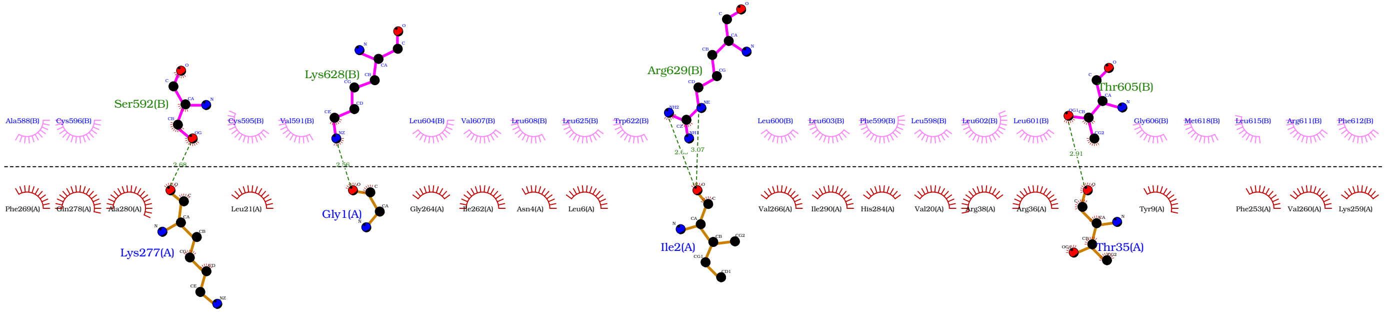
Ser592(B)
Lys628(B)
Arg629(B)
Thr605(B)
Ala588(B)
Cys596(B)
Cys595(B)
Val591(B)
Leu604(B)
Val607(B)
Leu608(B)
Leu625(B)
Trp622(B)
Leu600(B)
Leu603(B)
Phe599(B)
Leu598(B)
Leu602(B)
Leu601(B)
Gly606(B)
Met618(B)
Leu615(B)
Arg611(B)
Phe612(B)
2.68
2.56
3.07
2.91
Phe269(A)
Gln278(A)
Ala280(A)
Leu21(A)
Gly264(A)
Ile262(A)
Asn4(A)
Leu6(A)
Val266(A)
Ile290(A)
His284(A)
Val20(A)
Arg38(A)
Arg36(A)
Tyr9(A)
Phe253(A)
Val260(A)
Lys259(A)
Lys277(A)
Gly1(A)
Ile2(A)
Thr35(A)

G

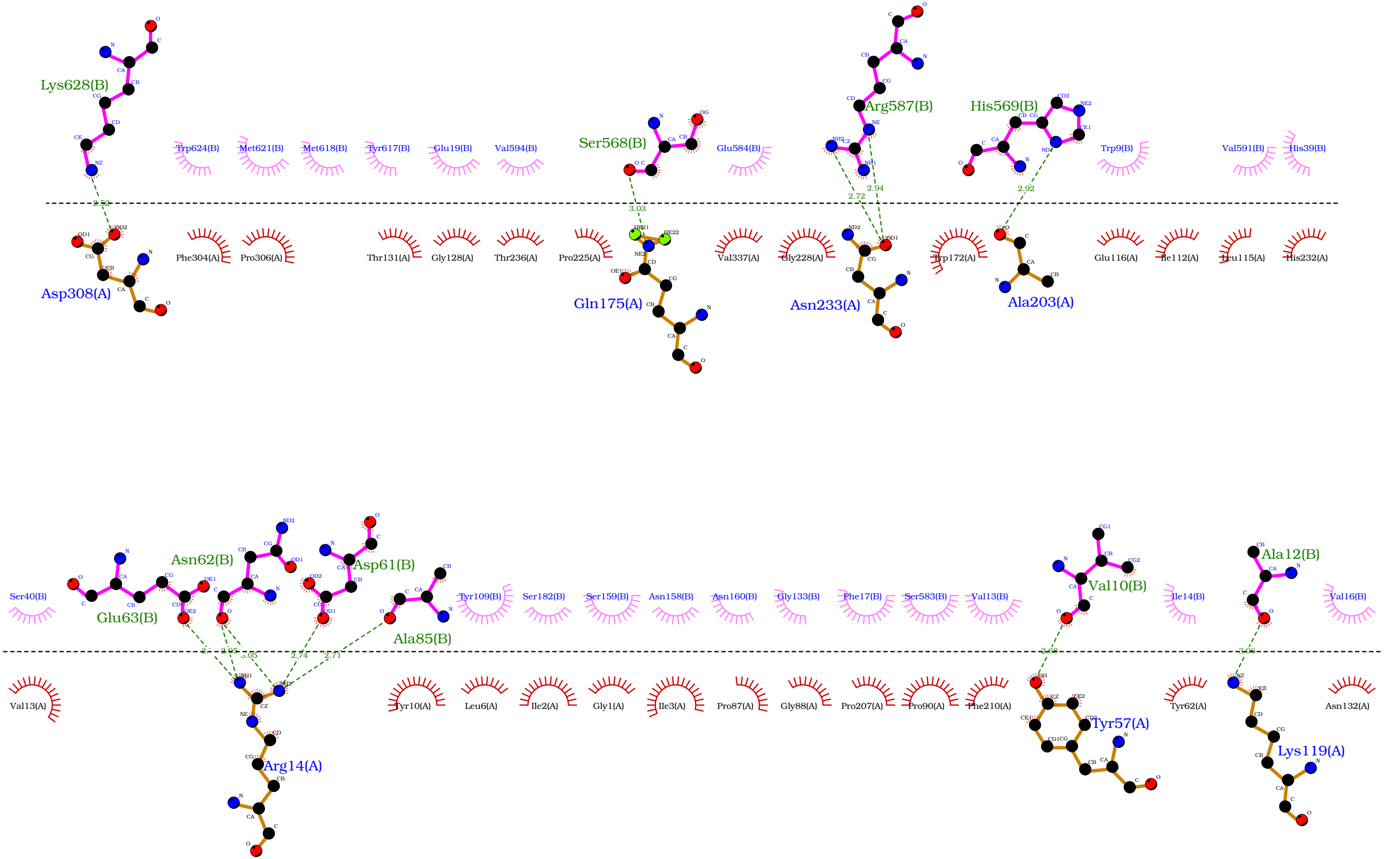
Lys628(B)
Trp624(B)
Met621(B)
Met618(B)
Tyr617(B)
Glu19(B)
Val594(B)
Ser568(B)
Glu584(B)
Arg587(B)
His569(B)
Trp9(B)
Val591(B)
His39(B)
3.03
2.72
2.94
2.92
Asp308(A)
Phe304(A)
Pro306(A)
Thr131(A)
Gly128(A)
Thr236(A)
Pro225(A)
Gln175(A)
Val337(A)
Gly228(A)
Asn233(A)
Trp172(A)
Ala203(A)
Glu116(A)
Ile112(A)
Leu115(A)
His232(A)
Ser40(B)
Glu63(B)
Asn62(B)
Asp61(B)
Ala85(B)
Tyr109(B)
Ser182(B)
Ser159(B)
Asn158(B)
Asn160(B)
Gly133(B)
Phe17(B)
Ser583(B)
Val13(B)
Val10(B)
Ile14(B)
Ala12(B)
Val16(B)
2.95
3.05
2.74
2.71
Val13(A)
Arg14(A)
Tyr10(A)
Leu6(A)
Ile2(A)
Gly1(A)
Ile3(A)
Pro87(A)
Gly88(A)
Pro207(A)
Pro90(A)
Phe210(A)
Tyr57(A)
Tyr62(A)
Lys119(A)
Asn132(A)

A

Figure S4

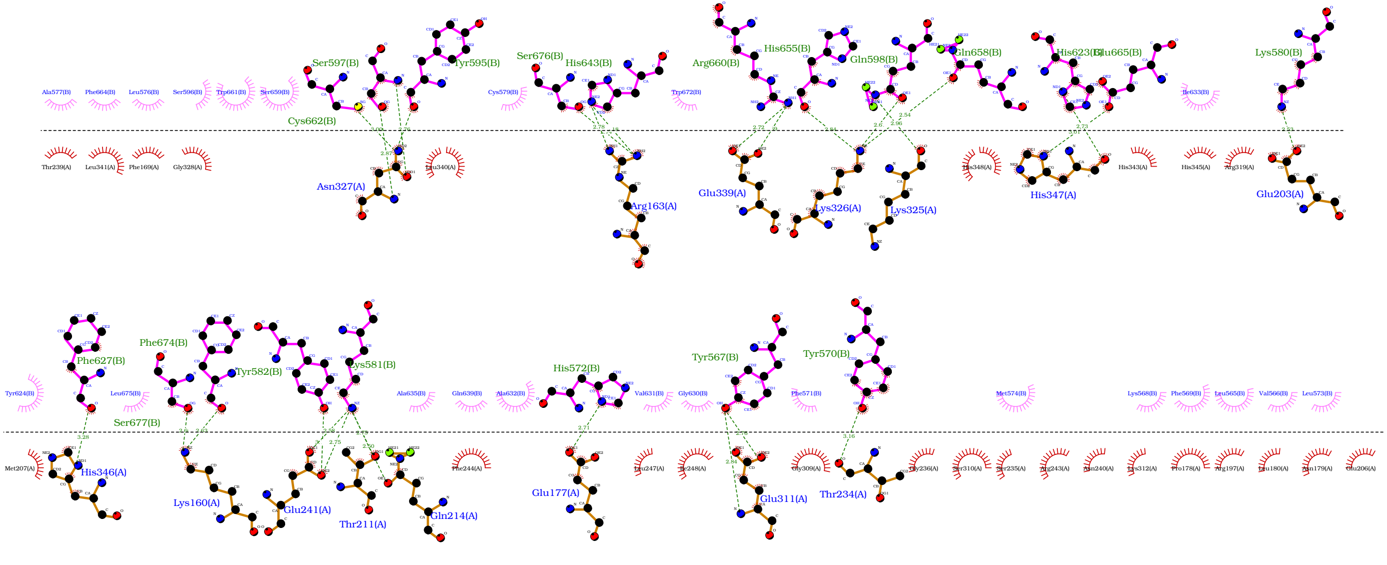
Ser597(B)
Tyr595(B)
Cys662(B)
Ala577(B)
Phe664(B)
Leu576(B)
Ser596(B)
Trp661(B)
Ser659(B)
Asn327(A)
Leu340(A)
Thr239(A)
Leu341(A)
Phe169(A)
Gly328(A)
2.87
2.76
Ser676(B)
His643(B)
Cys579(B)
Trp672(B)
Arg163(A)
2.78
Arg660(B)
His655(B)
Gln598(B)
Gln658(B)
His623(B)
Glu665(B)
Lys580(B)
Ile633(B)
Glu339(A)
Lys326(A)
Lys325(A)
His348(A)
His347(A)
His343(A)
His345(A)
Arg319(A)
Glu203(A)
2.72
2.6
2.96
2.54
2.73
Phe674(B)
Phe627(B)
Tyr582(B)
Lys581(B)
Ser677(B)
Tyr624(B)
Leu675(B)
Ala635(B)
Gln639(B)
Ala632(B)
His572(B)
Val631(B)
Gly630(B)
Tyr567(B)
Tyr570(B)
Phe571(B)
Met574(B)
Lys568(B)
Phe569(B)
Leu565(B)
Val566(B)
Leu573(B)
3.28
2.75
2.71
2.84
3.16
Met207(A)
His346(A)
Lys160(A)
Glu241(A)
Thr211(A)
Gln214(A)
Phe244(A)
Glu177(A)
Leu247(A)
Ile248(A)
Gly309(A)
Glu311(A)
Thr234(A)
Gly236(A)
Ser310(A)
Ser235(A)
Arg243(A)
Asn240(A)
Lys312(A)
Pro178(A)
Arg197(A)
Leu180(A)
Asn179(A)
Glu206(A)

B

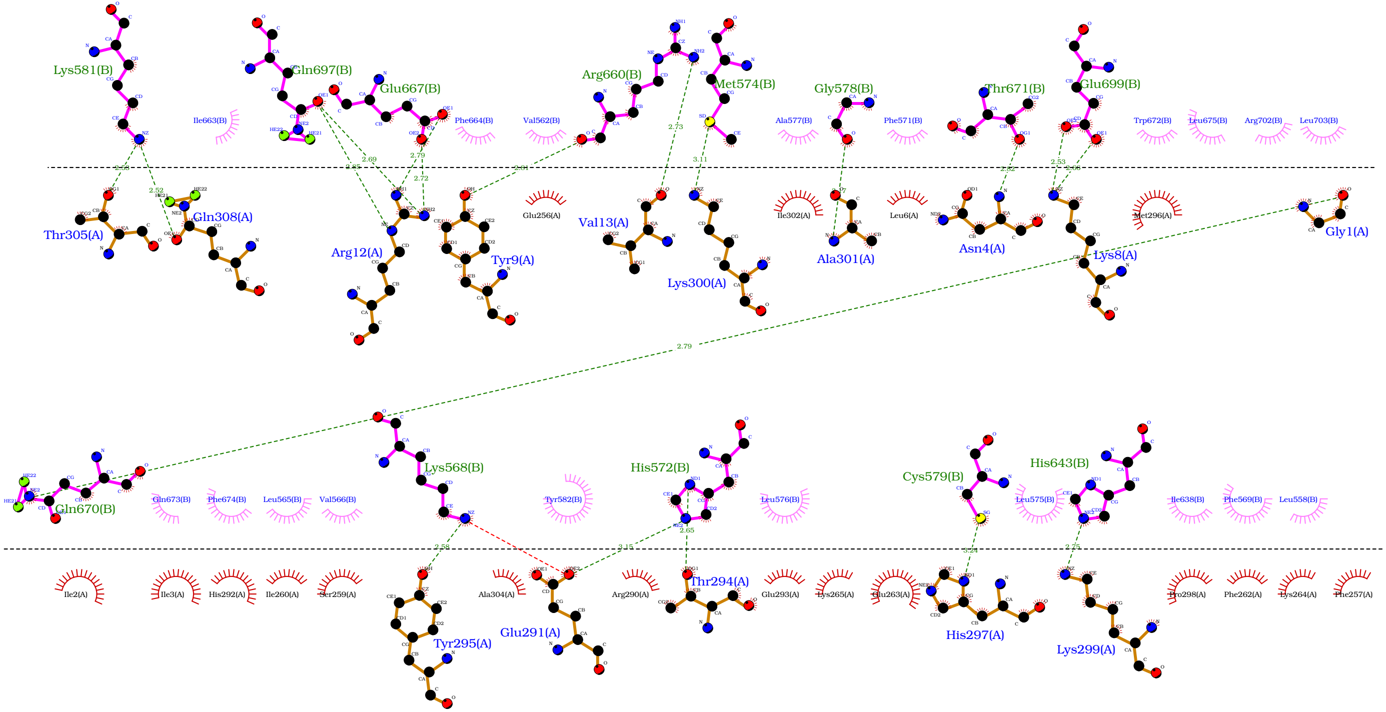
Lys581(B)
Gln697(B)
Glu667(B)
Arg660(B)
Met574(B)
Gly578(B)
Thr671(B)
Glu699(B)
Ile663(B)
Phe664(B)
Val562(B)
Ala577(B)
Phe571(B)
Trp672(B)
Leu675(B)
Arg702(B)
Leu703(B)
Thr305(A)
Gln308(A)
Arg12(A)
Tyr9(A)
Glu256(A)
Val13(A)
Lys300(A)
Ile302(A)
Ala301(A)
Leu6(A)
Asn4(A)
Met296(A)
Lys8(A)
Gly1(A)
2.79
Gln670(B)
Gln673(B)
Phe674(B)
Leu565(B)
Val566(B)
Lys568(B)
Tyr582(B)
His572(B)
Leu576(B)
Cys579(B)
His643(B)
Leu575(B)
Ile638(B)
Phe569(B)
Leu558(B)
Ile2(A)
Ile3(A)
His292(A)
Ile260(A)
Ser259(A)
Tyr295(A)
Ala304(A)
Glu291(A)
Arg290(A)
Thr294(A)
Glu293(A)
Lys265(A)
Glu263(A)
His297(A)
Lys299(A)
Pro298(A)
Phe262(A)
Lys264(A)
Phe257(A)

C

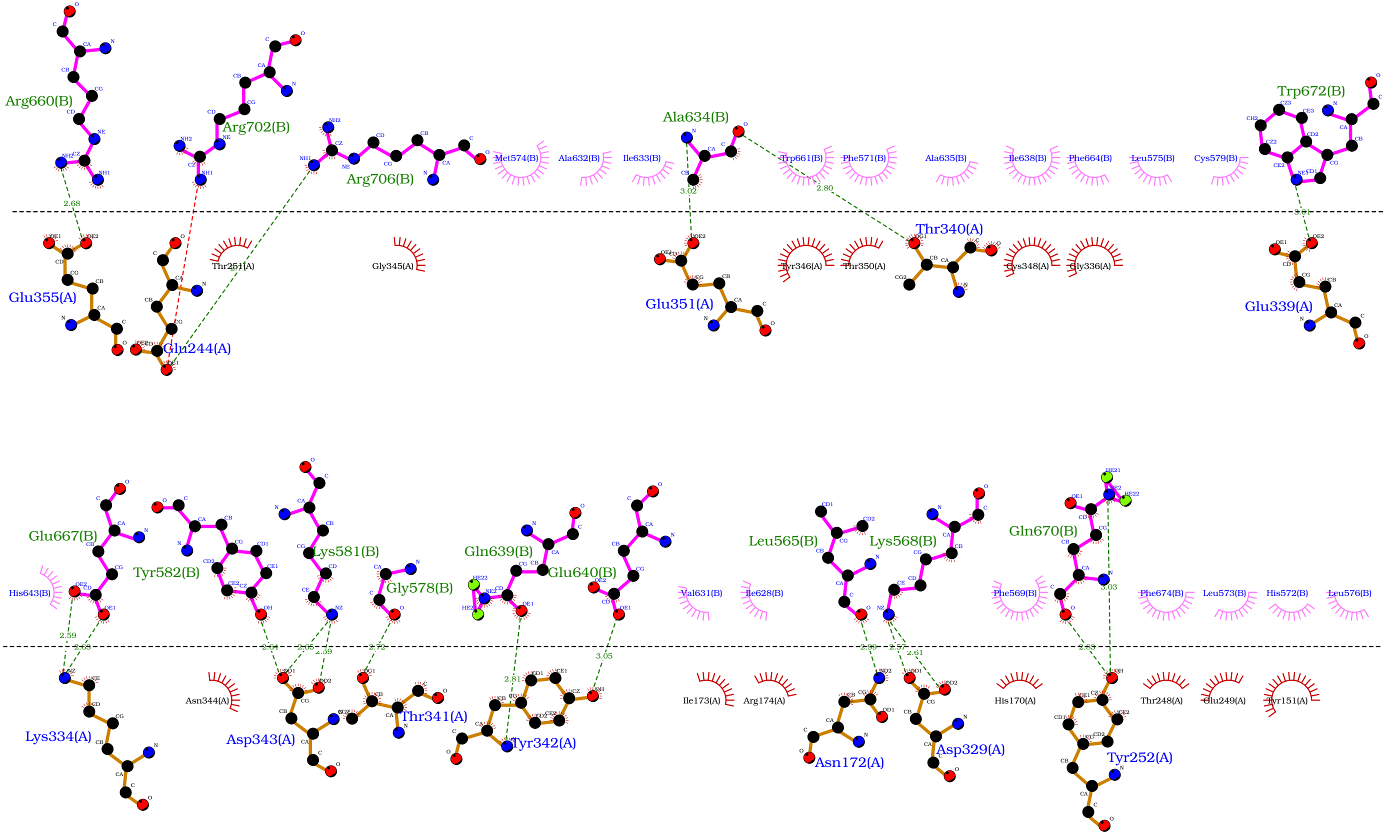

Arg660(B)
Arg702(B)
Arg706(B)
Ala634(B)
Trp672(B)
Met574(B)
Ala632(B)
Ile633(B)
Trp661(B)
Phe571(B)
Ala635(B)
Ile638(B)
Phe664(B)
Leu575(B)
Cys579(B)
2.68
3.02
2.80
Thr251(A)
Gly345(A)
Thr340(A)
Tyr346(A)
Thr350(A)
Cys348(A)
Gly336(A)
Glu355(A)
Glu244(A)
Glu351(A)
Glu339(A)
Glu667(B)
Tyr582(B)
Lys581(B)
Gly578(B)
Gln639(B)
Glu640(B)
Leu565(B)
Lys568(B)
Gln670(B)
His643(B)
Val631(B)
Ile628(B)
Phe569(B)
Phe674(B)
Leu573(B)
His572(B)
Leu576(B)
2.59
2.81
3.05
2.61
3.03
Asn344(A)
Lys334(A)
Asp343(A)
Thr341(A)
Tyr342(A)
Ile173(A)
Arg174(A)
His170(A)
Thr248(A)
Glu249(A)
Tyr151(A)
Asn172(A)
Asp329(A)
Tyr252(A)

D

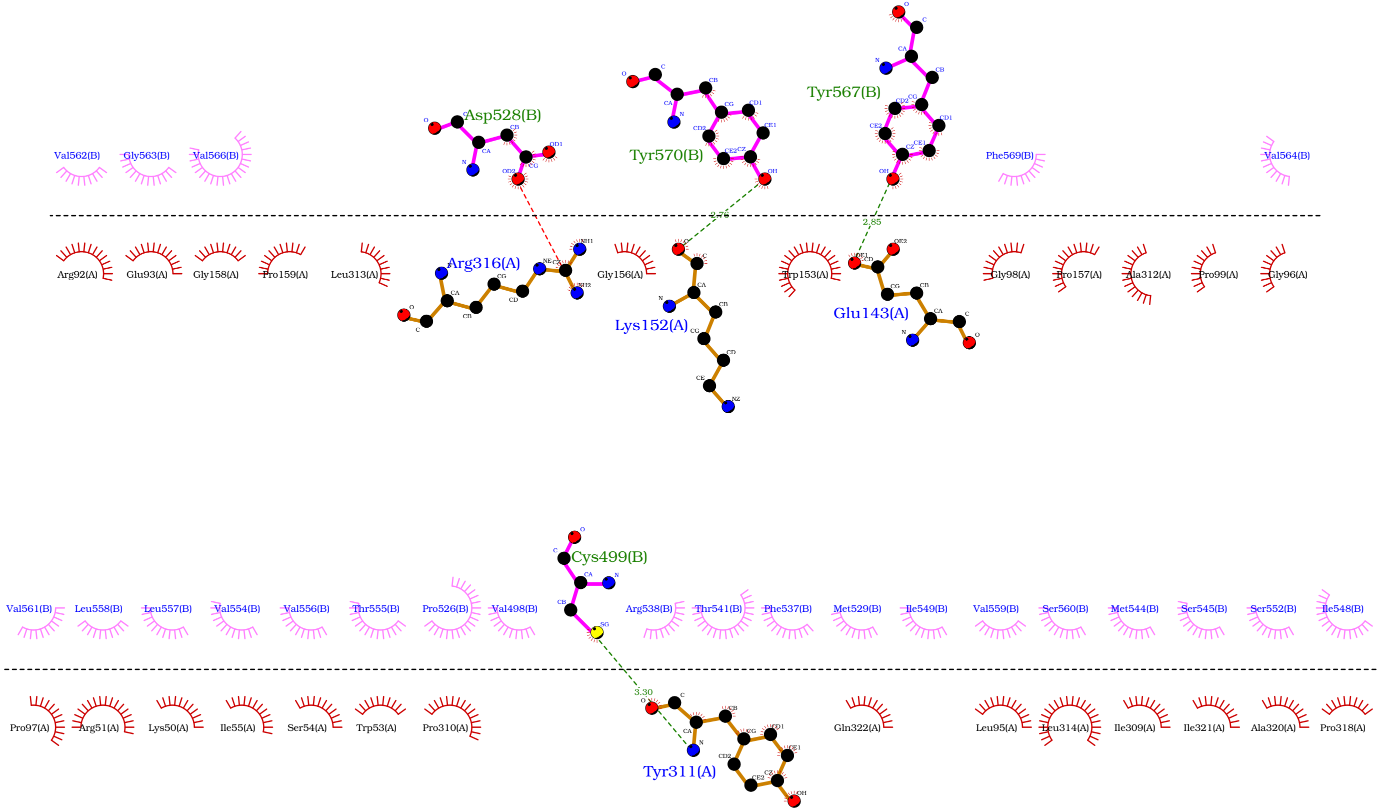
Asp528(B)
Tyr570(B)
Tyr567(B)
Val562(B)
Gly563(B)
Val566(B)
Phe569(B)
Val564(B)
2.76
2.85
Arg92(A)
Glu93(A)
Gly158(A)
Pro159(A)
Leu313(A)
Arg316(A)
Gly156(A)
Lys152(A)
Trp153(A)
Glu143(A)
Gly98(A)
Pro157(A)
Ala312(A)
Pro99(A)
Gly96(A)
Cys499(B)
Val561(B)
Leu558(B)
Leu557(B)
Val554(B)
Val556(B)
Thr555(B)
Pro526(B)
Val498(B)
Arg538(B)
Thr541(B)
Phe537(B)
Met529(B)
Ile549(B)
Val559(B)
Ser560(B)
Met544(B)
Ser545(B)
Ser552(B)
Ile548(B)
3.30
Pro97(A)
Arg51(A)
Lys50(A)
Ile55(A)
Ser54(A)
Trp53(A)
Pro310(A)
Tyr311(A)
Gln322(A)
Leu95(A)
Leu314(A)
Ile309(A)
Ile321(A)
Ala320(A)
Pro318(A)

E

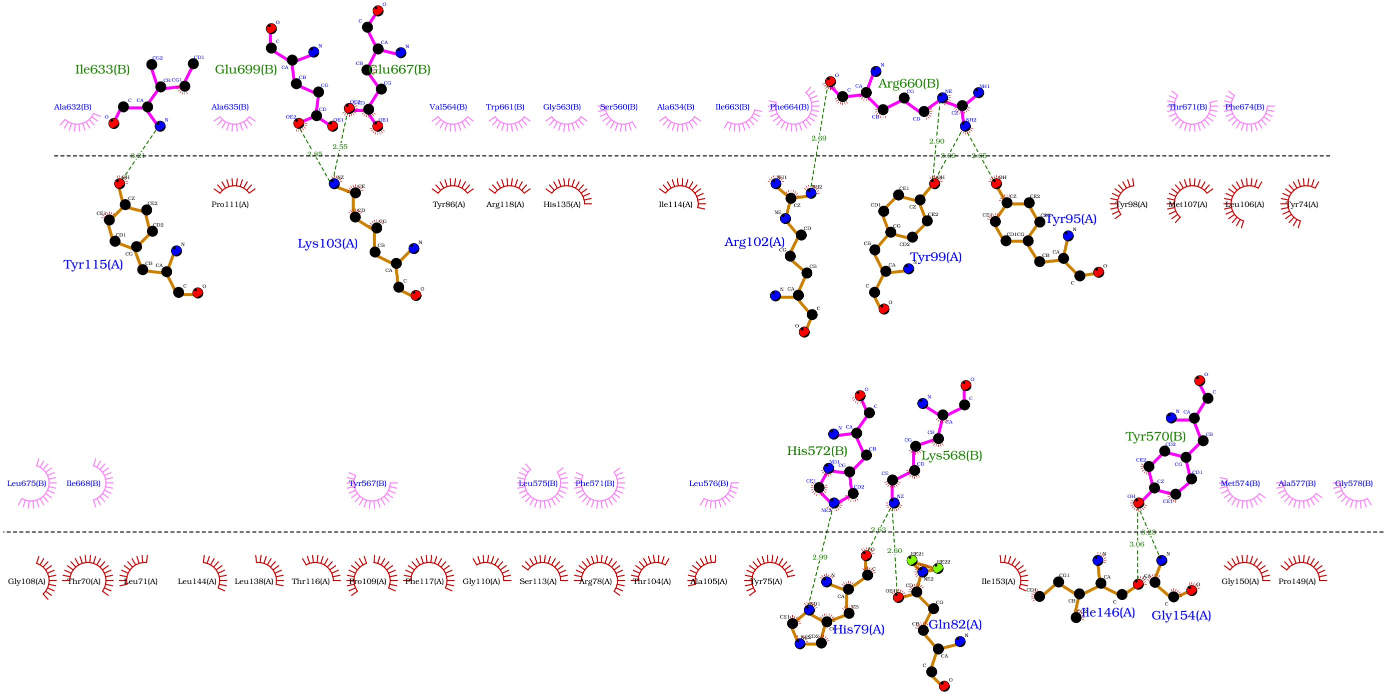

Ile633(B)
Glu699(B)
Glu667(B)
Arg660(B)
Ala632(B)
Ala635(B)
Val564(B)
Trp661(B)
Gly563(B)
Ser560(B)
Ala634(B)
Ile663(B)
Phe664(B)
Thr671(B)
Phe674(B)
2.69
2.90
2.85
2.55
Pro111(A)
Tyr86(A)
Arg118(A)
His135(A)
Ile114(A)
Tyr98(A)
Met107(A)
Leu106(A)
Tyr74(A)
Tyr115(A)
Lys103(A)
Arg102(A)
Tyr99(A)
Tyr95(A)
His572(B)
Lys568(B)
Tyr570(B)
Leu675(B)
Ile668(B)
Tyr567(B)
Leu575(B)
Phe571(B)
Leu576(B)
Met574(B)
Ala577(B)
Gly578(B)
2.99
2.60
3.06
Gly108(A)
Thr70(A)
Leu71(A)
Leu144(A)
Leu138(A)
Thr116(A)
Pro109(A)
Phe117(A)
Gly110(A)
Ser113(A)
Arg78(A)
Thr104(A)
Ala105(A)
Tyr75(A)
Ile153(A)
Gly150(A)
Pro149(A)
His79(A)
Gln82(A)
Ile146(A)
Gly154(A)

F

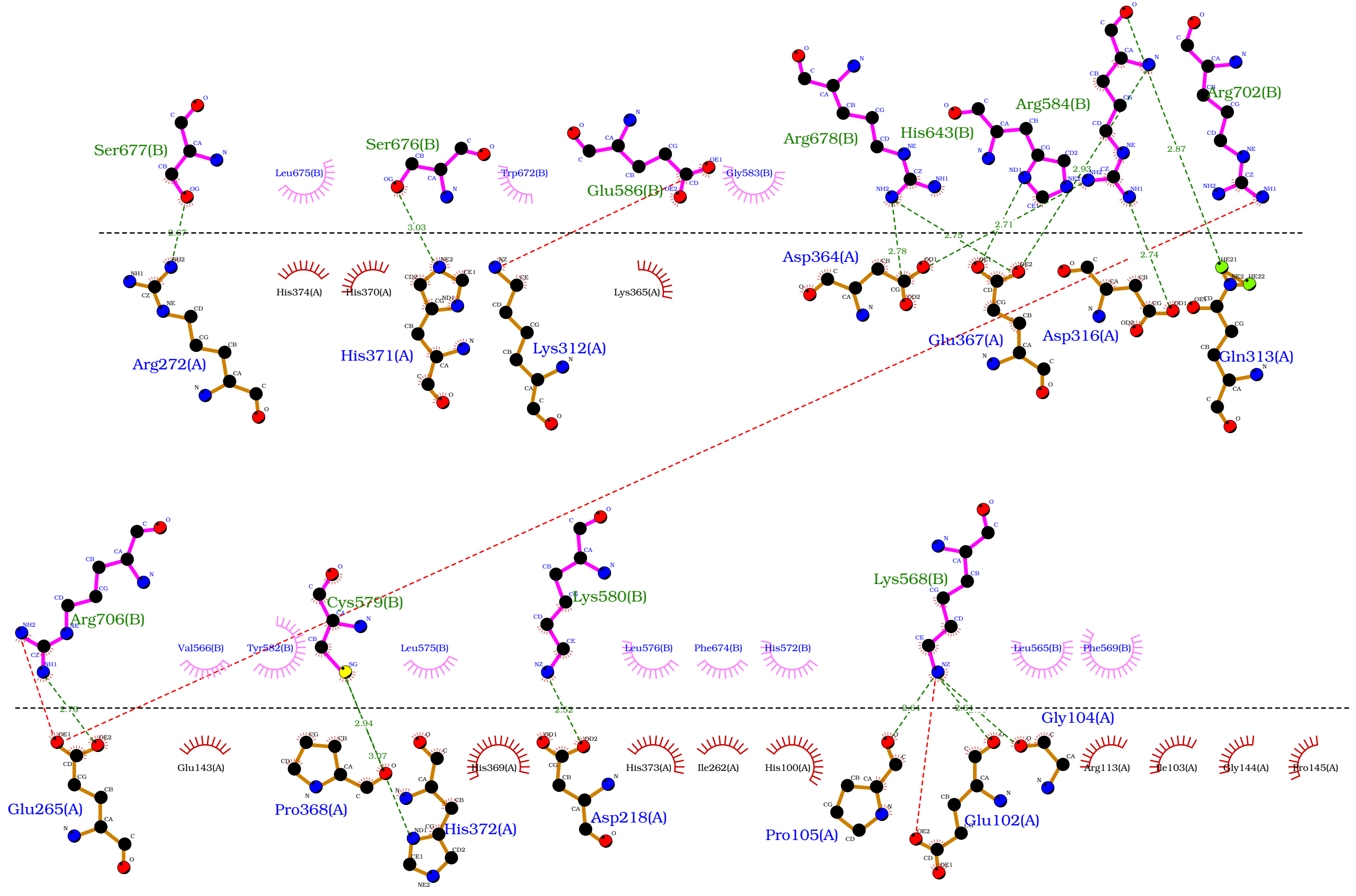

Ser677(B)
Leu675(B)
Ser676(B)
Trp672(B)
Glu586(B)
Gly583(B)
Arg678(B)
His643(B)
Arg584(B)
Arg702(B)
2.67
3.03
2.78
2.75
2.71
2.93
2.74
2.87
Arg272(A)
His374(A)
His370(A)
His371(A)
Lys312(A)
Lys365(A)
Asp364(A)
Glu367(A)
Asp316(A)
Gln313(A)
Arg706(B)
Val566(B)
Tyr582(B)
Cys579(B)
Leu575(B)
Lys580(B)
Leu576(B)
Phe674(B)
His572(B)
Lys568(B)
Leu565(B)
Phe569(B)
2.78
2.94
3.07
2.52
2.64
2.64
Glu265(A)
Glu143(A)
Pro368(A)
His372(A)
His369(A)
Asp218(A)
His373(A)
Ile262(A)
His100(A)
Pro105(A)
Glu102(A)
Gly104(A)
Arg113(A)
Ile103(A)
Gly144(A)
Pro145(A)

G

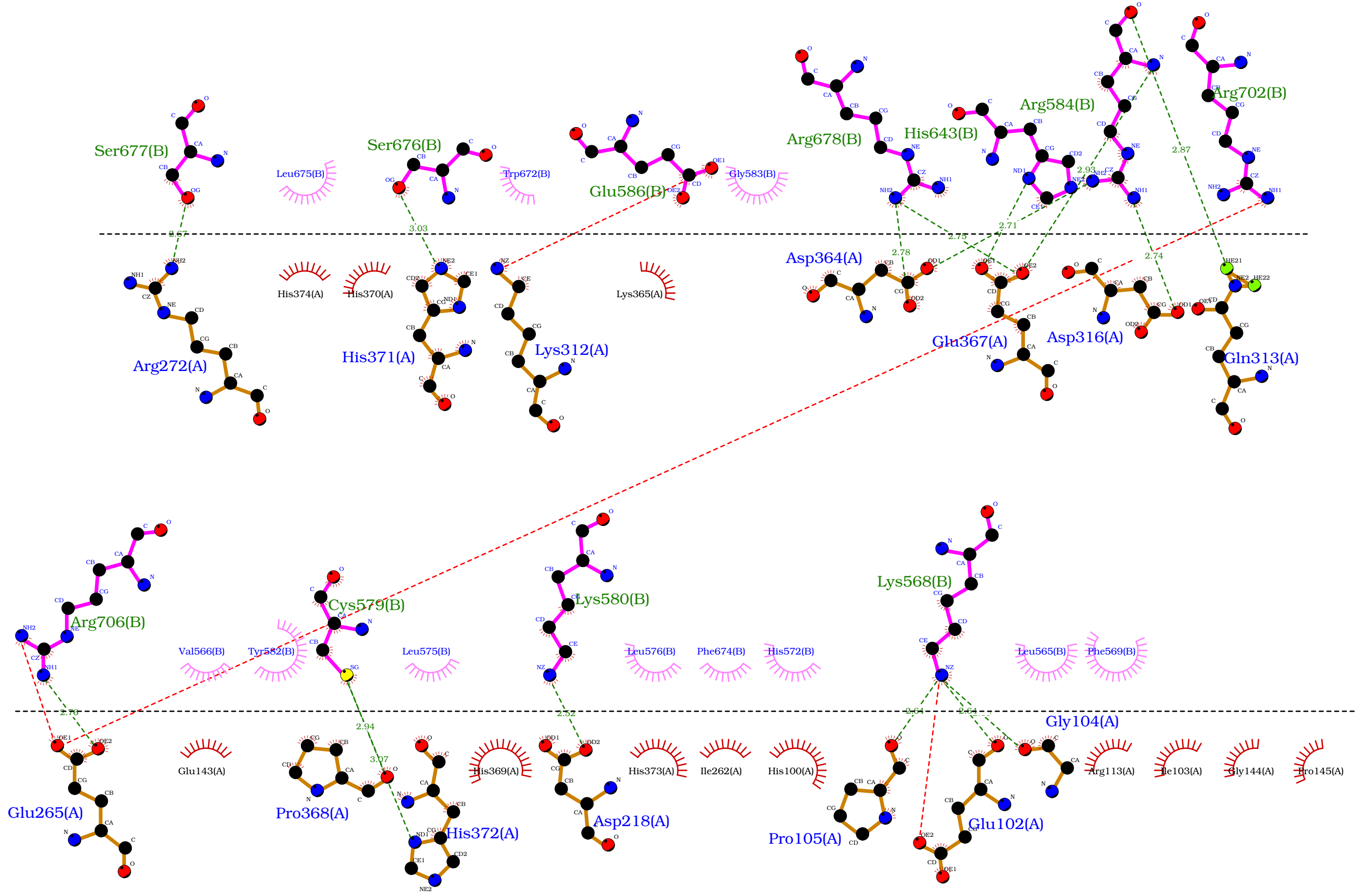

Ser677(B)
Leu675(B)
Ser676(B)
Trp672(B)
Glu586(B)
Gly583(B)
Arg678(B)
His643(B)
Arg584(B)
Arg702(B)
2.67
3.03
2.78
2.75
2.71
2.93
2.87
2.74
Arg272(A)
His374(A)
His370(A)
His371(A)
Lys312(A)
Lys365(A)
Asp364(A)
Glu367(A)
Asp316(A)
Gln313(A)
Arg706(B)
Val566(B)
Tyr582(B)
Cys579(B)
Leu575(B)
Lys580(B)
Leu576(B)
Phe674(B)
His572(B)
Lys568(B)
Leu565(B)
Phe569(B)
2.79
2.94
3.07
2.52
2.64
Glu265(A)
Glu143(A)
Pro368(A)
His369(A)
His372(A)
Asp218(A)
His373(A)
Ile262(A)
His100(A)
Pro105(A)
Glu102(A)
Gly104(A)
Arg113(A)
Ile103(A)
Gly144(A)
Pro145(A)
